# Prolonged anesthesia induces neuroinflammation and complement-mediated microglial synaptic elimination involved in neurocognitive dysfunction and anxiety-like behaviors

**DOI:** 10.1186/s12916-022-02705-6

**Published:** 2023-01-05

**Authors:** Feng Xu, Linlin Han, Yafeng Wang, Daling Deng, Yuanyuan Ding, Shuai Zhao, Qingtong Zhang, Lulin Ma, Xiangdong Chen

**Affiliations:** 1 Department of Anesthesiology, Union Hospital, Tongji Medical College, Huazhong University of Science and Technology, Wuhan, 430022 Hubei China; 2 Department of Anesthesiology, Affiliated Jinling Hospital, Medical School of Nanjing University, Nanjing, 210018 China

**Keywords:** Sevoflurane-induced neurotoxicity, Perioperative neurocognitive disorders, Microglia, Complement, Neuroinflammation, Synaptic elimination

## Abstract

**Background:**

Perioperative neurocognitive disorders (PND) with a high incidence frequently occur in elderly surgical patients closely associated with prolonged anesthesia-induced neurotoxicity. The neuromorphopathological underpinnings of anesthesia-induced neurotoxicity have remained elusive.

**Methods:**

Prolonged anesthesia with sevoflurane was used to establish the sevoflurane-induced neurotoxicity (SIN) animal model. Morris water maze, elevated plus maze, and open field test were employed to track SIN rats’ cognitive behavior and anxiety-like behaviors. We investigated the neuropathological basis of SIN through techniques such as transcriptomic, electrophysiology, molecular biology, scanning electron microscope, Golgi staining, TUNEL assay, and morphological analysis. Our work further clarifies the pathological mechanism of SIN by depleting microglia, inhibiting neuroinflammation, and C1q neutralization.

**Results:**

This study shows that prolonged anesthesia triggers activation of the NF-κB inflammatory pathway, neuroinflammation, inhibition of neuronal excitability, cognitive dysfunction, and anxiety-like behaviors. RNA sequencing found that genes of different types of synapses were downregulated after prolonged anesthesia. Microglial migration, activation, and phagocytosis were enhanced. Microglial morphological alterations were also observed. C1qa, the initiator of the complement cascade, and C3 were increased, and C1qa tagging synapses were also elevated. Then, we found that the “Eat Me” complement pathway mediated microglial synaptic engulfment in the hippocampus after prolonged anesthesia. Afterward, synapses were remarkably lost in the hippocampus. Furthermore, dendritic spines were reduced, and their genes were also downregulated. Depleting microglia ameliorated the activation of neuroinflammation and complement and rescued synaptic loss, cognitive dysfunction, and anxiety-like behaviors. When neuroinflammatory inhibition or C1q neutralization occurred, complement was also decreased, and synaptic elimination was interrupted.

**Conclusions:**

These findings illustrated that prolonged anesthesia triggered neuroinflammation and complement-mediated microglial synaptic engulfment that pathologically caused synaptic elimination in SIN. We have demonstrated the neuromorphopathological underpinnings of SIN, which have direct therapeutic relevance for PND patients.

**Supplementary Information:**

The online version contains supplementary material available at 10.1186/s12916-022-02705-6.

## Background

Sevoflurane-induced neurotoxicity (SIN) closely associated with perioperative neurocognitive disorders (PND) is an increasing complication after anesthesia, with an alarming rate in elderly surgical patients. SIN is triggered by prolonged sevoflurane anesthesia or a high dose of sevoflurane exposure, which is characterized by progressive cognitive dysfunction, anxiety, and sensory decline, causing a prolonged hospital stay, postoperative recovery delay, and an enormous clinical and socioeconomic burden. SIN is an essential topic in perioperative medicine, but it is understudied, which is a concern.

A number of clinical and animal studies have established an association between PND and inhalation anesthesia-induced neurotoxicity (sevoflurane) [1–3]. The existing evidence on SIN recognizes the critical role played by neuroinflammation in the hippocampus [4–6]. However, the neuromorphopathological underpinnings of cognitive impairment under the influence of neuroinflammation remain elusive. To date, many published studies have mainly focused on examining signaling pathways or molecules involved in regulating neuroinflammation in SIN [4, 7, 8]. Few studies have systematically investigated the neuronal morphology and synaptic structure directly related to learning and memory.

Synapses are the neural basis of learning and memory formation. Synaptic elimination caused by aberrant microglial activation drives some central nervous system diseases, such as Alzheimer’s disease, frontotemporal dementia, and forgetting, which are involved in complement activation and microglia-mediated synaptic pruning [9–11]. Depletion of microglia or C1q can inhibit synaptic pruning and synaptic elimination and ameliorate disease development [9, 11]. During the progression of SIN, microglial activation and neuroinflammation occur. Whether this notion about microglia mediating synaptic elimination also holds true for the etiology of SIN is unclear.

We employed RNA sequencing (RNA-seq) and molecular biological techniques to deeply explore the neuromorphopathological factors involved in SIN and evaluated the impact of microglia-mediated synaptic pruning on SIN. The findings of this study converge on biological processes associated with synaptic signaling and imply a specific pathological mechanism of synapse loss, which contributes to SIN development.

## Methods

### Animal groups

The animal protocol of the present study was approved by the Institutional Animal Care and Use Committee at Tongji Medical College, Huazhong University of Science and Technology. All animal experiments were performed in compliance with animal care guidelines. Thirteen- to eighteen-month-old male Sprague Dawley (SD) rats were commercially obtained from Biont (Wuhan, China). To verify our hypothesis, this study conducted four animal experiments. First, we used 3% sevoflurane in 100% oxygen to induce the SIN model, and the SD rats were randomly divided into two groups, the control and sevoflurane (Sevo) groups. SD rats in the Sevo group were exposed to 3% sevoflurane in 100% oxygen for 4 h, and rats in the control group were exposed to 100% oxygen for 4 h.

In the second experiment, rats were fed AIN-76A chow supplemented with PLX3397, a selective CSF1R (Colony-stimulating factor 1 receptor) inhibitor that has been demonstrated to readily cross the blood-brain barrier (BBB) and deplete microglia by oral delivery in rat chows [12, 13]. Vehicle chow (AIN-76A chow) was used to feed rats in the control group. Then, the rats used for SIN establishment by sevoflurane exposure were assigned to two groups: the Sevo-AIN76A and Sevo-PLX3397 groups.

In the third experiment, rats were intraperitoneally injected with meloxicam, a nonsteroidal anti-inflammatory drug (NSAID) that has been shown to effectively reduce hippocampal inflammation [14]. Intraperitoneal injection of normal saline (NS) was used as a carrier control. Then, following SIN establishment by sevoflurane exposure, these rats were assigned to two groups: the Sevo-NS and Sevo-meloxicam groups.

In the fourth experiment, rats received intracerebroventricular (i.c.v) injection of 10 μl C1q neutralizing antibody (clone JL-1, GTX54404, GeneTex) or IgG2b in PBS, which were administered at 1 h after anesthesia end. According to previous work, the i.c.v. injection site was located as: AP (anteroposterior) = −1.2 mm, ML (mediolateral) = 1.8 mm, and DV (dorsoventral) = 4.0 mm [15]. Then, following SIN establishment by sevoflurane exposure, these rats were assigned to two groups: the Sevo-IgG and Sevo-C1q groups.

### The SIN rat model

Focusing on examining the SIN, we used a long time and 3% sevoflurane in 100% oxygen for rat anesthesia, which has been reported to effectively trigger cognitive impairment in previous studies [2, 16, 17]. SD rats were exposed to 3% sevoflurane in 100% oxygen for 4 h in an anesthetizing box in which the concentration of sevoflurane and carbon dioxide and the temperature were detected through an anesthesia monitor. After sevoflurane exposure, the rats were immediately transferred to a resuscitation chamber filled with fresh oxygen. The air temperature was set in the range of 23–25 °C. Rats in the control group were exposed to the carrier gas (100% oxygen) for 4 h.

### PLX3397 formulation in rat chow

PLX3397 (TOPSCIENCE, China) was purchased to eliminate microglia in the brain. Following the methods of a previous study, PLX3397 was formulated in AIN-76A chow at a concentration of 300 mg/kg chow [18]. PLX3397-supplemented chow was produced by Jiangsu Xietong, Inc. (Nanjing, China). Based on published studies, rats were fed either AIN-76A or AIN-76A chow supplemented with PLX3397 (300 mg/kg) for 21 days [18, 19]. Then, after feeding for 21 days, the number of microglia and the expression of iba1 (a microglial marker) were measured to estimate the efficacy of microglia elimination.

### Meloxicam injection

Meloxicam injection (Kangdien, China) was used to inhibit neuroinflammation in the SIN rats. A dose of 2.5 mg/kg was proven to show a protective anti-inflammatory effect [20]. Meloxicam injection was administered three times: at the start of anesthesia, at the end of anesthesia, and 1 h later, based on a previous study [20]. Inflammatory cytokines were detected to evaluate the anti-inflammatory effect.

### Tests of cognition

The Morris water maze (MWM) test, a widely used measure of learning and memory, was used to evaluate cognitive impairment, as described in a published study [21]. During neurobehavioral experiments in this study, experimenters were all blind to group assignment and outcome assignment. Briefly, SD rats underwent 5 days of training, including three periods (swimming training with a visible platform, swimming training with a hidden platform, and a probe trial). On day 1, the rats received swimming training four times a day with a visible platform in one quadrant of the pool. From days 2 to 5, the rats received swimming training four times a day with a hidden platform in the same quadrant of the pool. During the training period, if the rats found the platform within 60 s, they were allowed to stand on the platform for 5 s. Rats that failed to find the platform within 60 s were placed on the platform for 20 s. In the probe trial, the platform was removed from the pool. Then, the time spent in the platform quadrant and the number of platform quadrant crossings were measured for 60 s. The probe trial was conducted on the first and third days after anesthesia. Since the MWM test showed no difference on day 3 after sevoflurane exposure, we ended the MMW test on the third day after anesthesia.

Considering the influence of brain trauma after intracerebroventricular injection on rats’ motor ability as well as water in MWM causing wound infection, we used contextual fear conditioning (CFC) to test memory ability. The CFC chamber was composed of a white plastic chamber with a stainless steel grid floor. Each rat was taken from its home cage, and each animal was allowed to explore for 3 min for habituation. Each rat was conditioned with A 30-s, 80-dB, 4500-Hz tone co-terminated with a 2-s, 1.5-mA footshock, with an inter-trial interval of 1 min, for 4 min. Next 2 days, each rat was placed back in the CFC chamber for 3 min. The freezing behaviors of rats were recorded by Xeye software (Zhongshi Technology, China). The memory performance was estimated by the percentage of freezing time.

### Exploratory activity and anxiety-like behavior

Exploratory activity and anxiety-like behavior were evaluated by the open field test (OFT) and elevated plus maze (EPM) test [22]. In the OFT, an open field apparatus was used, and the central zone was a square 20 cm away from the wall of the apparatus. Rats were placed in the central zone and allowed autonomous exploration. The time spent in the central zone and the number of crossings in the central zone were recorded for 5 min. In the EPM test, an apparatus mainly consists of two closed arms and two opened arms. The closed arms in the apparatus were enclosed by a black wall. Each rat was placed in the central region of the EPM apparatus facing the opened arm. The time of staying in different arms and the number of entering different arms were measured for 5 min. Since the cognitive tests showed no difference on day 3 after anesthesia, we focused only on the behavioral change on day 1 after sevoflurane exposure. The OFT and EPM tests were performed on the first day after anesthesia.

### Electrode placement and hippocampal local field potential (LFP) recordings

The rats were administrated with continuous 3% sevoflurane on an animal anesthesia mask and then fixed onto a stereotaxic frame (RWD, China). After disinfecting the scalp and applying additional analgesia using bupivacaine, a longitudinal incision along the midline was made to expose the bregma. One PFA-coated LFP electrode (stainless steel wire) was positioned in the left hippocampus (AP −4.5 mm; ML −3.0 mm; DV −4.0 mm), and a reference electrode (silver wire) was placed over the left cerebellum. Subsequently, all electrodes were skull-secured with dental cement. Before the LFP recordings, at least 7 days were allowed for the animals to recover from the surgery.

Rats were individually positioned in a custom-made 20-L airtight chamber. An inlet and an outlet were placed on the opposite sidewalls for gas delivery and discharge. EEG signals were continuously sampled (1 kHz/s) and bandpass-filtered at 0.1–500 Hz using PowerLab 8/35 (ADInstruments, Australia). Moreover, LabChart 8.0 software (ADInstruments, Australia) was used for data recording and analysis. The LFP was sequentially recorded in the air for 30 min as a control and in sevoflurane (3%) for 30 min as a test. Recordings ended when the rats emerged from anesthesia. Only the data recorded during relatively quiet conditions were used for analysis to avoid interference caused by vigorous exercise.

### Burst suppression identification and analysis

The raw data were first bandpass-filtered at 0–45 Hz and exported into MATLAB R2016b (Mathworks, MA, USA) for processing, as described in our previous study [23]. In the present study, a suppression signal is defined as an amplitude limited within ± 15 μV for at least 200 ms, and bursts are defined as epochs between suppression events. Suppression and burst are, respectively, given a value of 1 and 0 to produce a binary time series. The burst suppression ratio (BSR) was calculated as the percentage of suppression time of each 1 min binary series [12].

### Brain tissue collection

Since the cognitive tests showed a significant difference on the first day after anesthesia, rats were deeply anesthetized with sevoflurane on the first day after the SIN model establishment for collecting tissues. In brief, after opening up the skull, brain tissue was collected for molecular biology experiments, and a glass dissecting needle was employed to peel off the brain tissue. We used a saline flush and paraformaldehyde fixation, and brain slices were prepared for staining.

### RNA sequencing (RNA-seq)

Hippocampal tissues were collected and immediately sent to BGI Genomics for RNA-seq processing (Beijing Genomics Institute, BGI, China). The cDNA library establishment was in compliance with the BGI standard procedures. Briefly, the fragment was end-repaired, dA-tailed, adaptor-ligated, and then subjected to a 4-cycle PCR program. The libraries were sequenced based on the BGI protocols for RNA-seq on the Illumina HiSeq 2500 platform using the 50 bp pair-end sequencing strategy. Transcript expression levels were evaluated by using fragments per kilobase per million reads (FPKM). In terms of functional enrichment analysis, all DEGs (differentially expressed genes) were mapped to terms in the Kyoto Encyclopedia of Genes and Genomes (KEGG) and Gene Ontology (GO) databases. Dor. Tom from BGI (a web-based multiomics visualization tool: https://biosys.bgi.com/#/report/mrna/en/help) was used to analyze and visualize the results of RNA-seq.

### Real-time polymerase chain reaction (RT-PCR)

Following the manufacturer’s instructions, an EntiLink™ 1st Strand cDNA Synthesis Kit (ELK Biotechnology, China) was used to prepare the cDNA of the hippocampus. RT-PCR was implemented by using an EnTurbo™ SYBR Green PCR SuperMix kit (ELK Biotechnology, China) and a StepOne™ Real-Time PCR machine (Thermo Fisher Scientific, USA). The reaction system was prepared according to the instructions of the EnTurbo™ SYBR Green PCR SuperMix kit. The cycling parameters were set at 95 °C for 3 min, then 40 cycles at 95 °C for 10 s, followed by 58 °C for 30 s and 72 °C for 30 s. All gene primer pairs are reported in Additional file 1: Table. S1.

### Enzyme-linked immunosorbent assay (ELISA)

Proinflammatory cytokines (TNF-α, IL-6, and IL-1β) and C1q in the hippocampus were measured with ELISA kits (Bioswamp, China). According to the manufacturer’s instructions, an ELISA was employed to detect the TNF-α, IL-6, IL-1β, and C1q levels in the protein supernatant. A microplate reader (Thermo Fisher Scientific, USA) with absorption at 450 nm was employed to measure the absorbance values of the samples, and the proinflammatory cytokine concentration was detected based on the standard curve produced.

### Western blotting

Western blotting was performed as described in our previous work in detail [24, 25]. The hippocampus was minced into homogenates in a lysis buffer with protease and phosphatase inhibitors. The supernatants were collected after the homogenates were centrifuged at 4 °C at 12,000 rpm for 15 min. A BCA protein assay Kit (Biosharp, China) was used to detect the protein concentration. Then, the supernatants were mixed with 5×SDS-PAGE (sodium dodecyl sulfate–polyacrylamide gel electrophoresis) loading buffer (Biosharp, China). Hippocampal proteins were separated by 8–10% SDS-PAGE and transferred to PVDF membranes (Millipore, USA). Then, the PVDF membranes were immersed in the indicated primary antibodies overnight. The primary antibodies were as follows: anti-iba1 (1/1000, ab178846, Abcam, UK), anti-PSD95 (1/1000, 36233S, Cell Signaling Technology, USA), anti-Synapsin-1 (SYN1) (1/1000, 5297S, Cell Signaling Technology, USA), anti-Synaptophysin (SYP) (1/1000, 36406S, Cell Signaling Technology, USA), anti-C1qa (1/1000, sc-58920, Santa Cruz Biotechnology, USA), anti-CD68 (1/1000, ab125212, Abcam, UK), anti-Phospho-NF-κB p65 (1/1000, 3039S, Cell Signaling Technology, USA), anti-Caspase-3 (1/1000, 9662S, Cell Signaling Technology, USA), anti-bcl-2 (1/1000, ab196495, Abcam, USA), anti-bax (1/1000, 2772S, Cell Signaling Technology, USA), and anti-β-actin (1/5000, ab170325, Abcam, UK). Afterward, the PVDF membranes were incubated with the appropriate horseradish peroxidase-labeled secondary antibodies, including anti-rabbit (1/10,000, Abcam, UK), anti-rat (1/10,000, Abcam, UK), and anti-mouse (1/10,000, Abcam, UK) antibodies. Then, ECL-A buffer and ECL-B buffer (Biosharp, China) were used for imaging. β-actin antibody was used as a loading control. The western blotting bands were quantified by using ImageJ software.

### Immunofluorescence staining

Brain slices were blocked with 5% BSA (bovine serum albumin) in PBS (phosphate-buffered saline) supplemented with 0.3% Triton X-100 for 1 h. Then, the brain slices were incubated with the indicated primary antibodies, including iba1 (1/200, ab178846, Abcam, UK), PSD95 (1/200, 36233S, Cell Signaling Technology, USA), PSD95 (1/200, 3450S, Cell Signaling Technology, USA), SYN1 (1/200, 5297S, Cell Signaling Technology, USA), SYP (1/200, 36406S, Cell Signaling Technology, USA), C1qa (1/50, sc-58920, Santa Cruz Biotechnology, USA), C1q (1/100, GTX54404, GeneTex, USA), CD68 (1/100, ab125212, Abcam, UK), C-fos (1/100, AF5354, Affinity Biosciences, China), Phospho-NF-κB p65 (1/100, 3033T, Cell Signaling Technology, USA), and CX3CR1 (1/50, sc-377227, Santa Cruz Biotechnology, USA). Two primary antibodies from different species were coincubated with the same brain slice for bimolecular staining. After washing, the brain slices were immersed in the appropriate secondary antibodies, including goat anti-rabbit IgG antibody conjugated with Alexa Fluor 488 (1/1000, Abcam, UK), goat anti-rabbit IgG antibody conjugated with Alexa Fluor 594 (1/1000, Abcam, UK), goat anti-mouse IgG antibody conjugated with Alexa Fluor 488 (1/1000, Abcam, UK), goat anti-mouse IgG antibody conjugated with Alexa Fluor 594 (1/1000, Abcam, UK), and donkey anti-rat IgG antibody conjugated with Alexa Fluor 488 (1/1000, Abcam, UK) for 1 h at RT (Room temperature) in a dark room. Finally, the sections were placed in a mounting medium with DAPI-Aqueous, Fluoroshield (Abcam, UK) and then imaged under a fluorescence microscope or a confocal laser scanning microscope.

### Hematoxylin and eosin (HE) staining

HE staining was employed to observe neuronal morphology and inflammatory cell infiltration. Brain slices were stained by using an H&E kit (Solarbio, China) according to the manufacturer’s instructions and then imaged under a microscope.

### Nissl staining

Nissl staining was used to detect Nissl bodies in the neurons, which is considered an indicator of neural damage [25]. Nissl staining was performed in accordance with the instructions of the Nissl staining kit (Solarbio, China), and the brain slices were imaged under a microscope. Then, morphological alterations in the neurons and the number of Nissl bodies in the hippocampus were observed by a microscopy.

### TUNEL assay

Neuronal apoptosis was evaluated by TUNEL assay, as described in our previous work [24]. The TUNEL assay was performed using an In Situ Cell Death Detection Kit (Roche, Switzerland) according to the manufacturer’s instructions. Fluorescence microscopy was employed to identify neuronal apoptosis in the whole brain slice.

### Scanning electron microscope (SEM)

Briefly, rats were perfused with 0.1 M sodium cacodylate buffer containing 4% paraformaldehyde (PFA) and 2.5% glutaraldehyde, and immediately, 1-mm coronal sections were collected. The CA1 region of the hippocampus was microdissected and fixed in 2.5% glutaraldehyde in 0.1 M sodium cacodylate buffer at 4 °C for 24 h. Then, hippocampal samples were sent to Jarvis Inc. (Wuhan, China) for staining and processing. The synaptic vesicles, synapses, and synaptic clefts were used as features to identify the synaptic structure.

### Golgi staining

An FD Rapid GolgiStain™ Kit (FD NeuroTechnologies, Inc., USA) was employed to examine the neuromorphopathological alterations. Golgi staining was performed according to the instructions. The rat brains were immersed in Golgi solution for 14 days in a dark room. The solution was changed every 2 days for 14 days. Then, the brain samples were sliced into 100-μm coronal sections using a vibrating microtome. The staining steps followed the instructions.

### Sholl analysis and quantitative morphology

We all know that cellular structure is fundamental to the execution of its functions. The Sholl plugin in ImageJ was used to describe and trace the morphologies [26, 27]. The Sholl plugin can perform analysis on grayscale images of isolated neurons or microglia. The Sholl plugin can skeletonize neurons or microglia. Then, it was used to analyze the color-coded dendrites and pseudopodia. Warner hues produced by the Sholl plugin indicated the number of intersections. The intersection mask was employed as the quantitative descriptor for comparisons between groups. The maximal intersection number was used to depict cell complexity and reflect the highest number of processes in the cell [28]. Solidity was defined as microglial cell area/convex area, which estimates the area of the cell and skeleton [29]. We used ImageJ to measure the morphological transformation in two-dimensional space.

### Statistics

The mean ± SD (standard deviation) was used to describe normally distributed data. The differences between the two groups were analyzed using *t*-tests or rank-sum tests. Paired *t*-tests were used to compare the results on the first and third days of the MWM. With regard to the RNA-seq results, |log2(fold change)| > 0.2 and adjusted *P* < 0.05 were set to achieve more DEGs [30], which have a potential association with SIN. The fold change of each gene was calculated by comparing the standardized read counts of one group to another group (fold change = standardized read counts of one group/standardized read counts of another group). A *P* < 0.05 was considered a significant difference. All data analyses and statistics were performed with Stata 16.0 (Stata Corporation, USA), and statistical charts were produced by GraphPad Prism 8.0 (GraphPad Software Inc., USA) and Stata 16.0.

## Results

### Prolonged anesthesia triggers cognitive dysfunction and anxiety-like behaviors

**Fig. 1.**
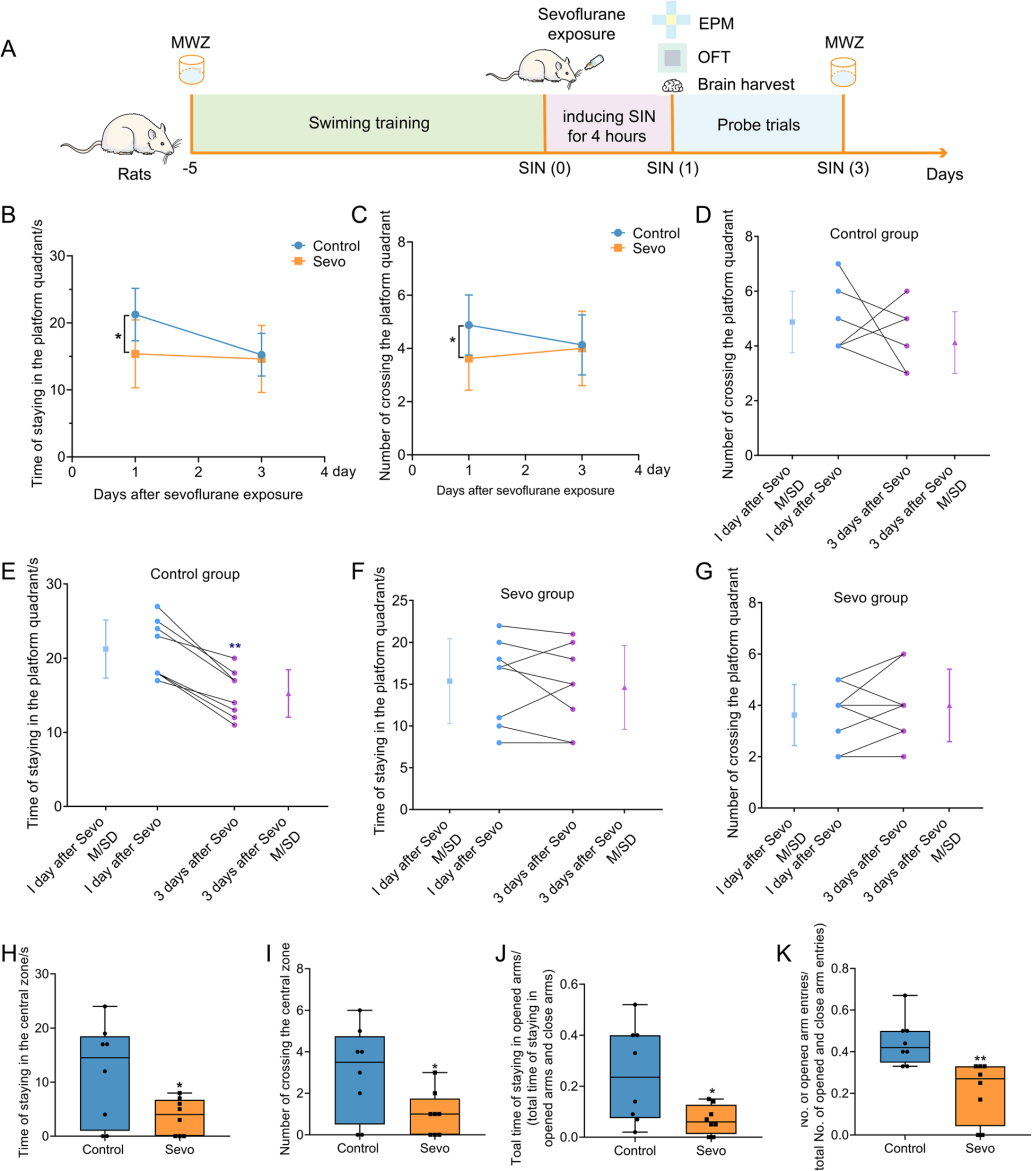
Prolonged anesthesia caused cognitive dysfunction and anxiety-like behaviors in rats. **A** The schedule of the first experiment. Rats underwent 5 days of swimming training in the MWM. Then, 3% sevoflurane was exposed to rats for 4 h to induce SIN rats. Probe trials were performed on the first and third days after sevoflurane exposure. On the first day after sevoflurane exposure, we harvested brain tissues. **B, C** Prolonged anesthesia by sevoflurane caused cognitive dysfunction in rats. It reduced the time and number of entering the platform quadrant of rats.  **D, E** The before-after with SD chart of the MWM test in the control group. **F, G** The before-after with SD chart of the MWM test in Sevo group. **H, I** In OFT, prolonged anesthesia caused anxiety-like behaviors in rats. It decreased the time and number of entering the central region of rats. **J, K** In EPM, prolonged anesthesia caused anxiety-like behaviors in rats. It decreased the number and time of entering opened arms of rats. Data was shown as Mean ± SD or Median ± interquartile range (IQR), with *P < 0.05 or *P < 0.001; *n* = 8 per group, Sevo group vs. control group

The schedule of the first experiment is displayed in Fig. 1 A. SIN rat models induced by sevoflurane exposure exhibited some neurobehavioral alterations, including cognitive dysfunction and anxiety-like behaviors. In the MWM test, on the first day after sevoflurane exposure, the time spent in the platform quadrant and the number of crossings in the platform quadrant by rats in the Sevo group were apparently decreased when compared with rats in the control group (Fig. 1 B, C). Then, on the third day after sevoflurane exposure, there was no difference in the MWM test between the two groups. We used before-after with an SD chart to examine each rat’s cognitive alteration per group. In the Sevo group, there was no difference in the MWM test between 1 day and 3 days after sevoflurane exposure (Fig. 1 F, G). However, in the control group, there was a significant difference in the time spent in the platform quadrant by the rats between 1 day and 3 days after sevoflurane exposure (Fig. 1 E). Thus, the cognitive decline of the control group rats over time may be the cause of the lack of a significant difference between the two groups on the third day after sevoflurane exposure. In the OFT, SIN rats spent less time staying in the central zone and less time entering the central zone (Fig. 1 H, I). Moreover, in the EPM test, we also found that the Sevo group rats had a decreased time and number of entries into the opened arms compared with the control group (Fig. 1 J, K). This evidence suggests that prolonged anesthesia triggers cognitive dysfunction and anxiety-like behaviors.

### Prolonged anesthesia induces neuroinflammation, upregulates the NF-κB inflammatory pathway, downregulates neuronal excitability, and has no effect on apoptotic signaling

**Fig. 2:**
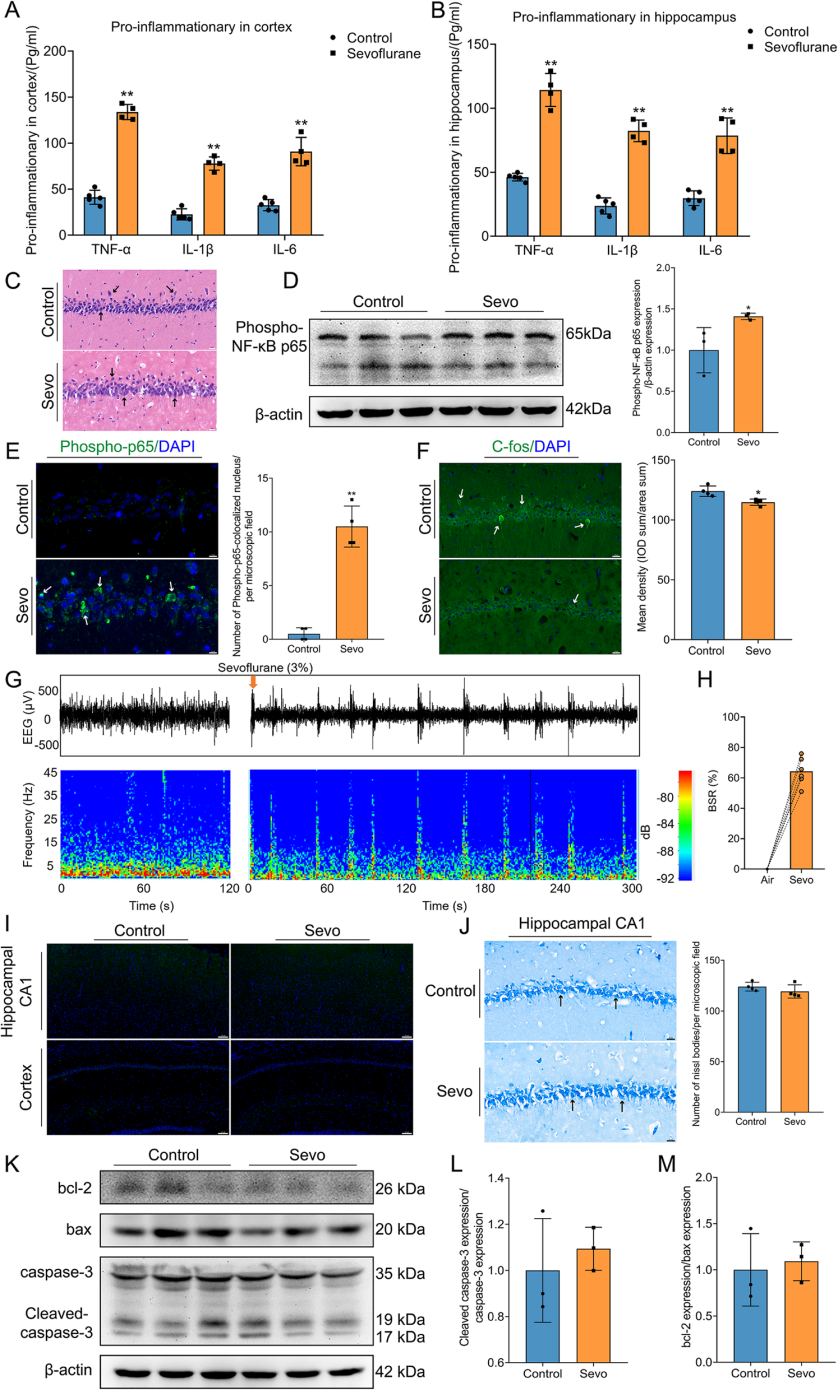
Prolonged anesthesia inducing neuroinflammation, upregulating NF-κB inflammatory pathway, downregulating neuronal excitability, and inactivating apoptotic signaling. **A, B** TNF-α, IL-1β, and IL-6 evidently increased in the cortex (**A**) and hippocampus (**B**) after prolonged anesthesia (*n* = 4 or 5 per group). **C** Effects of prolonged anesthesia on the morphological changes of neurons in the hippocampus. Scale bar = 20 μm. **D** Prolonged anesthesia activated NF-κB inflammatory pathway (*n* = 3 per group). **E** The number of Phospho-NF-κB P65-colocalized nuclei in the hippocampus (*n* = 4 per group). Scale bar = 10 μm. **F** Prolonged anesthesia inhibiting neuronal excitability marker C-fos expression in the hippocampal CA1 region (*n* = 4 per group). Scale bar = 20 μm. **G** Exhibiting representative EEG raw traces (upper) and power spectrograms (bottom) for the hippocampus. **H** Prolonged anesthesia triggered burst suppression in the hippocampus (*n* = 6 per group). The burst suppression ratio (BSR) was calculated as the percentage of suppression time of each 1 min binary series. **I** TUNEL staining in brain slices was negative after prolonged anesthesia. Scale bar = 100 μm. **J** The number of Nissl’s body (*n* = 4 per group). Scale bar = 20 μm. **K** Prolonged anesthesia had no effect on apoptotic pathways (*n* = 3 per group). **L**, **M** Quantification of Cleaved caspase-3/caspase-3 (**L**) and bcl-2/bax (**M**) levels normalized to β-actin. Data was shown as Mean ± SD, with *P < 0.05 or *P < 0.001; Sevo group vs. control group. Arrows represent positive cells or colocalization

Clinically, PND mainly occurs within 1 week after surgery and anesthesia [31]. Combined with the results of our behavioral experiments, we harvested the brain tissue of rats on the first day after sevoflurane exposure. We used ELISA to show that proinflammatory cytokines, including TNF-α, IL-1β, and IL-6, were increased in the cortex and hippocampus after sevoflurane exposure (Fig. 2 A, B). Furthermore, ELISA also showed increased peripheral inflammation after sevoflurane exposure (Additional file 2: Fig. S1). We also examined the NF-κB (nuclear factor kappa light-chain-enhancer of activated B cells) inflammatory pathway after sevoflurane exposure. Phospho-NF-κB p65 expression in the hippocampus was upregulated in the Sevo group compared with the control group (Fig. 2 D). After sevoflurane exposure, the translocation of Phospho-NF-κB p65 to the nucleus increased in the hippocampus (Fig. 2 E).

Then, we transferred our attention to the effect of sevoflurane exposure on neurons, the executive cellular basis of cognitive function. HE staining showed that the morphology of neurons in the two groups was intact in the hippocampus (Fig. 2 C). IF staining revealed that C-fos, as a parameter of neuronal excitability in the hippocampal CA1 region, was downregulated after sevoflurane exposure (Fig. 2 F). Similarly, EEG also showed burst suppression in the hippocampus. Figure 2 G shows representative EEG raw traces (upper) and power spectrograms (bottom) for the hippocampus, which indicate the transition to burst suppression after sevoflurane exposure. This change was also demonstrated by the significant increase in the BSR as presented in Fig. 2 H. This suppression of neural excitability led us to explore whether it is influenced by neuronal apoptosis. The TUNEL assay was negative without neuronal apoptosis in the hippocampal CA1 region and cortex after sevoflurane exposure (Fig. 2 I). Nissl staining also suggested that there was no difference in the number of Nissl bodies in the hippocampal CA1 region between the Sevo and control groups (Fig. 2 J). Furthermore, we also detected the expression of apoptotic signaling proteins, including cleaved caspase-3, caspase-3, bax, and bcl-2, in the hippocampus. There was no significant difference in cleaved caspase-3/caspase-3 or bcl-2/bax between the groups (Fig. 2 K–M). Thus, sevoflurane exposure produced no effect on apoptotic signaling. More importantly, prolonged anesthesia induces neuroinflammatory activation, activates the NF-κB inflammatory pathway, and downregulates neuronal excitability.

### Prolonged anesthesia causes microglial activation, increases proinflammatory phenotype markers, decreases anti-inflammatory phenotype markers, and alters microglial morphology in the hippocampus

**Fig. 3:**
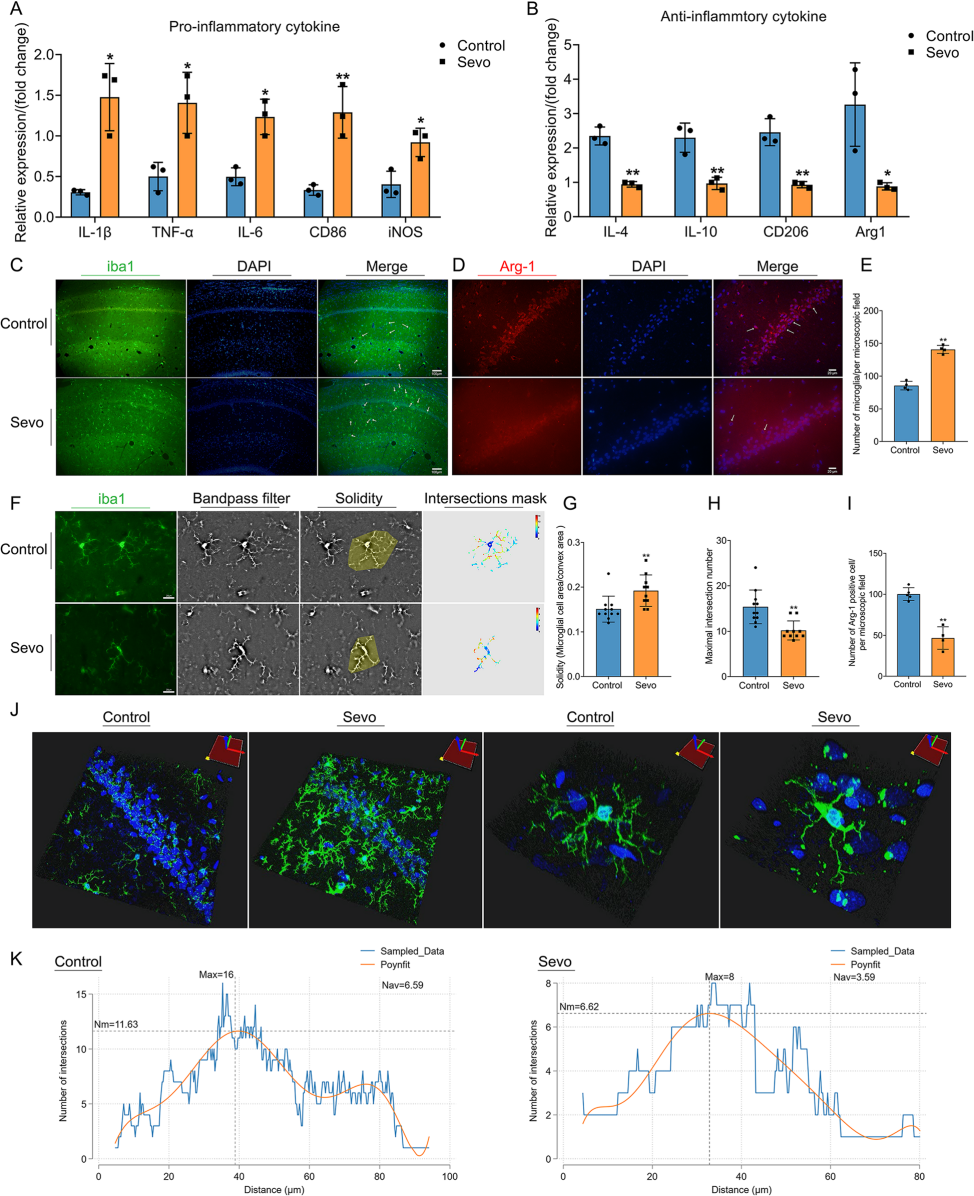
Prolonged anesthesia inducing microglial activation, increasing proinflammatory phenotype markers, decreasing anti-inflammatory phenotype markers, and altering microglial morphology in the hippocampus. **A** Prolonged anesthesia increased the expression of the mRNA of proinflammatory phenotype markers, including IL-1β, TNF-α, IL-6, CD86, and iNOS (*n* = 3 per group). **B** Prolonged anesthesia decreased the expression of the mRNA of anti-inflammatory phenotype markers, including IL-4, IL-10, CD206, and Arg1 (*n* = 3 per group). **C** Prolonged anesthesia increased the number of microglia in the hippocampal CA1 region. iba1 marked (green) microglia and DAPI (blue) marked the cell nucleus. Scale bar = 100 μm. **D** Prolonged anesthesia downregulated Arg1 expression in the hippocampus. Arg1 is marked in red. Scale bar = 20 μm. **E** The statistical chart of the number of microglia in both groups (*n* = 4 per group). **F** Prolonged anesthesia altered microglial morphology (*n* = 10 or 11 cells per group). We used the bandpass filter method in ImageJ to remove noise from IF staining and calculated microglial solidity and maximal intersection number (intersections mask). Scale bar = 20 μm. **G** Prolonged anesthesia increased microglial solidity. **H** Prolonged anesthesia decreased the maximal intersection number of microglia. **I** Prolonged anesthesia decreased Arg1 positive cells (*n* = 4 or 5 per group). **J** The 3-dimensional image reconstruction showed cell body of microglia was enlarged, and pseudopodia became fewer and shorter after prolonged anesthesia. In the CA1 region, microglia were accumulated with morphological alteration. **K** The polynomial fit curve uncovered that the number of intersections varies with distance from the nucleus. The place where the maximum intersection was located was closer to the cell body of microglia after prolonged anesthesia, as shown by the dotted line, which means a retraction of microglial pseudopodia. Nm = number of maximal intersection number; Nav = the average of all intersections; Polynomial fit curve = Poynfit. Data was shown as Mean ± SD, with *P < 0.05 or *P < 0.001; Sevo group vs. control group. Arrows represent positive cells

Microglia, as resident phagocytes in the central nervous system (CNS), are the first responders to stressors and they rapidly adjust their phenotype and undergo functional changes [32, 33], and they participate in the pathological process of SIN. After sevoflurane exposure, proinflammatory phenotype markers, including TNF-α mRNA, IL-1β mRNA, IL-6 mRNA, CD86 mRNA, and iNOS (inducible nitric oxide synthase) mRNA in the hippocampus, were all upregulated compared with those in the control group (Fig. 3 A). Moreover, anti-inflammatory phenotype markers, including IL-4 mRNA, IL-10 mRNA, CD206 mRNA, and Arg1 (arginase-1) mRNA in the hippocampus, were downregulated compared with the control group (Fig. 3 B). IF staining with iba1 (a marker for microglial activation) showed that the number of microglia increased in the hippocampus after sevoflurane exposure compared with the control group (Fig. 3 C, E), which implies microglial activation. Arg-1, as an anti-inflammatory marker, was downregulated in the hippocampal CA1 region after sevoflurane exposure compared with the control group (Fig. 3 D, I). We used Sholl analysis to identify microglial morphological alterations in the hippocampal CA1 region to confirm its activation and functional changes (Fig. 3 F). An intersection mask chart is shown to describe the spatial distribution and number of microglial pseudopodia (Fig. 3 F). The microglial solidity was increased, and the maximal intersection number was reduced after sevoflurane exposure (Fig. 3 G, H), which means that the microglial area was enlarged and the number of microglial pseudopodia was decreased. The intersection chart showed that the pseudopodia distribution was closer to the microglial cell body after sevoflurane exposure (Fig. 3 K), which illustrated the retraction of microglial pseudopodia. In addition, we also used a three-dimensional picture of microglia to observe microglial morphological alterations. Then, the cell body of the microglia enlarged and its pseudopodia decreased and became shorter, as shown in Fig. 3 J. These microglial morphological alterations were strongly induced by sevoflurane exposure.

### RNA-seq analysis found DEGs in the hippocampus were enriched in different synaptic pathways and synapse-related genes were downregulated after prolonged anesthesia

**Fig. 4:**
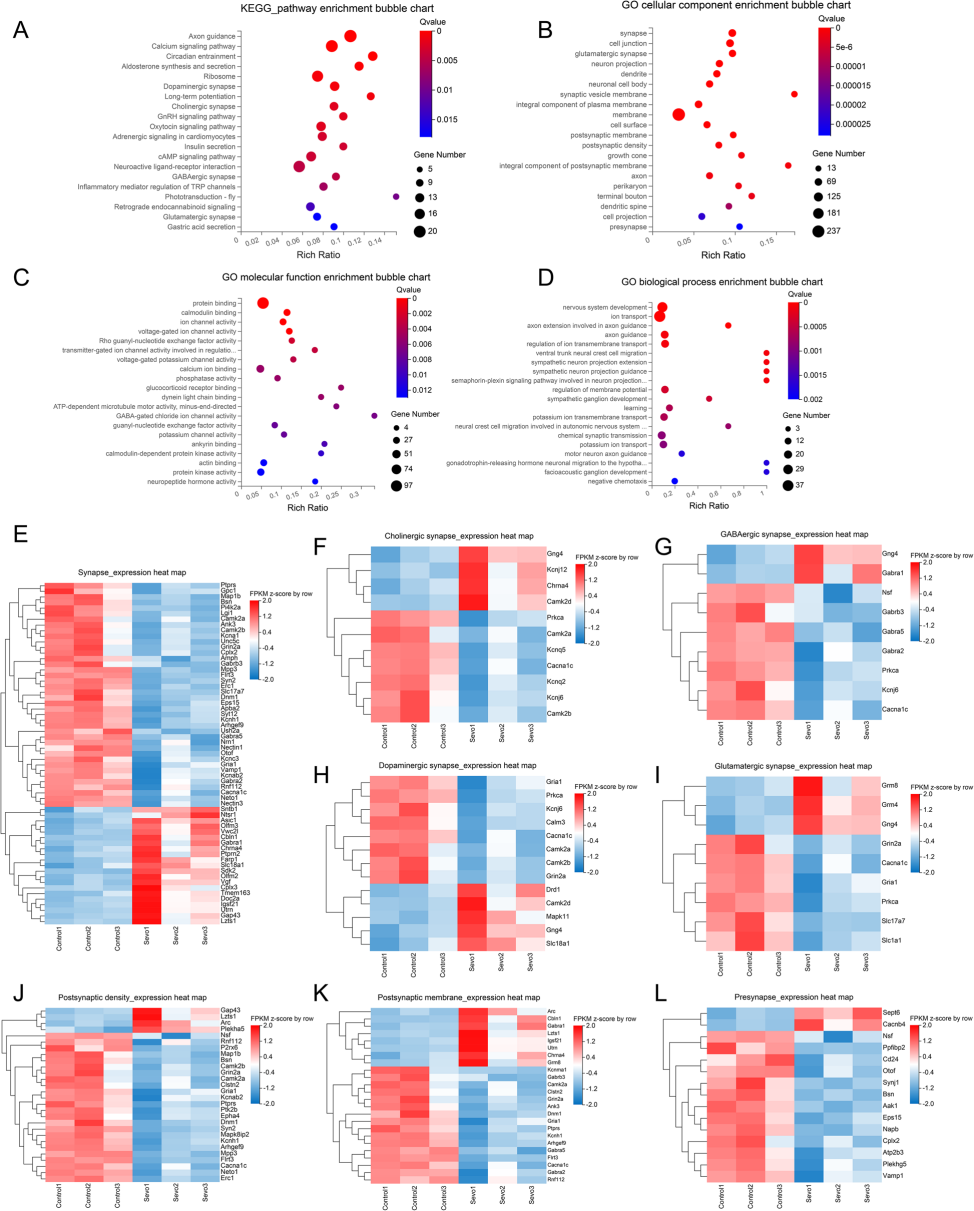
RNA-seq analysis indicated DEGs in the hippocampus were enriched in different synaptic pathways, and synapse-related genes were downregulated after prolonged anesthesia. **A** KEGG pathway enrichment bubble chart. **B** GO cellular component enrichment bubble chart. **C** GO molecular function enrichment bubble chart. **D** GO biological process enrichment bubble chart. From panels **A** to **D**, *X*-axis is the enrichment ratio (the ratio of the number of genes annotated to an entry in the selected gene set to the total number of genes annotated to the entry in the species), and *Y*-axis is KEGG pathway or GO term. The bubble size represents the number of genes annotated to the KEGG pathway or GO term. The color represents the enriched significance (*n* = 3 in each group). **E–I** Expression heat map of DEGs enriched in the synapse (**E**), cholinergic synapse (**F**), GABAergic synapse (**G**), dopaminergic synapse (**H**), and glutamatergic synapse (**I**). **J–L** Expression heat map of DEGs enriched in the postsynaptic density (**J**), postsynaptic membrane (**K**), and presynapse (**L**). From panels **E** to **L**, the horizontal axis is the *z*-score of the sample, and the vertical axis is the gene. The warmer the color block is, the higher the expression level is, and the colder the color block is, the lower the expression level is (*n* = 3 per group)

We used RNA-seq to screen for pathways and genes to identify the possible neuromorphopathological underpinnings causing SIN. Surprisingly, both KEGG pathway enrichment and GO enrichment analysis strongly suggested that most DEGs were associated with different synapses or synaptic components. In summary, the KEGG pathway enrichment chart showed that DEGs were primarily enriched in dopaminergic synapses, long-term potentiation, cholinergic synapses, GABAergic synapses, and glutamatergic synapses (Fig. 4 A). Furthermore, in GO cellular component enrichment analysis, the DEGs were mainly enriched in the synapse, glutamatergic synapse, synaptic vesicle membrane, postsynaptic membrane, postsynaptic density, integral components of the postsynaptic membrane, and presynapse (Fig. 4 B). GO molecular function enrichment analysis showed that the DEGs were primarily enriched in protein binding, calmodulin binding, ion channel activity, and GABA-gated chloride ion channel activity (Fig. 4 C). GO biological process enrichment analysis showed that the DEGs were mainly enriched in nervous system development, ion transport, axon guidance, learning, and chemical synaptic transmission (Fig. 4 D).

To identify the expression level of synapse-related genes, we employed a DEG expression heatmap to describe different types of synaptic genes. DEGs enriched in the synapse, glutamatergic, cholinergic, dopaminergic, and GABAergic synapses were mainly downregulated (Fig. 4 E–I). DEGs of synaptic components, including postsynaptic density, postsynaptic membrane, and presynapse, were also downregulated (Fig. 4 J–L). Hence, sevoflurane exposure resulted in the loss of synapse-related genes (e.g., syn2), and the DEGs were mainly enriched in different synapses, indicating synaptic elimination after prolonged anesthesia.

### Prolonged anesthesia enhances microglia phagocytosis and chemotaxis and causes synaptic elimination in the hippocampus by complement-mediated microglial pruning

**Fig. 5:**
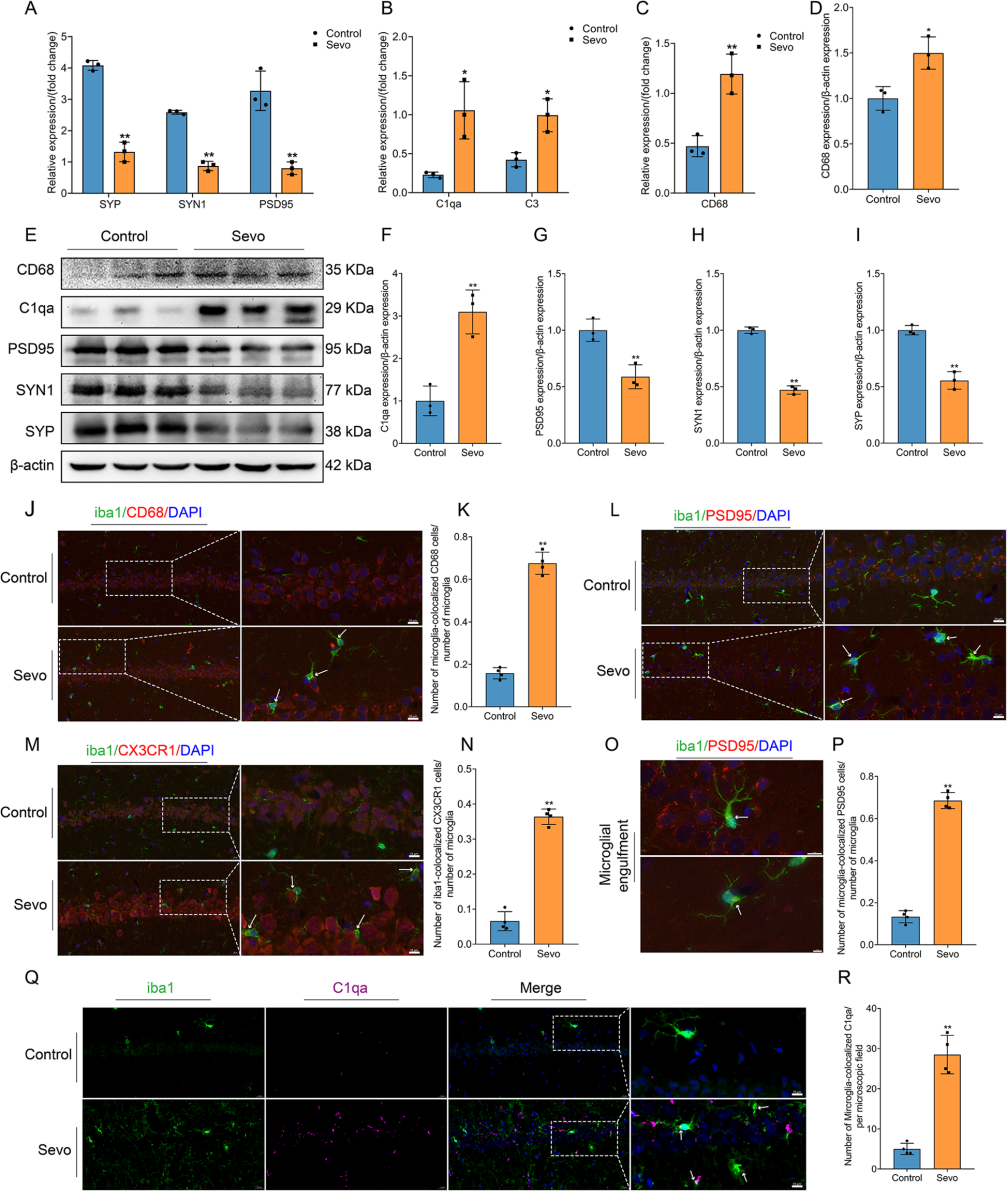
Prolonged anesthesia enhanced microglia phagocytosis and chemotaxis and then triggered microglial synaptic engulfment resulting in synaptic loss. **A** Prolonged anesthesia decreased the expression of the mRNA of synaptic markers, including SYP, SYN1, and PSD95 (*n* = 3 per group). **B** Prolonged anesthesia increased the expression of the mRNA of complement markers, including C1qa and C3 (*n* = 3 per group). **C** Prolonged anesthesia increased the expression of the mRNA of the microglial phagocytosis marker (CD68) (*n* = 3 per group). **D** Prolonged anesthesia increased the expression of the microglial phagocytosis marker (CD68) at the protein level (*n* = 3 per group). **E** Immunoblotting band of CD68, C1qa, PSD95, SYN1, SYP, and β-actin (*n* = 3 per group). **F** Prolonged anesthesia increased the expression of C1qa. **G–I** Prolonged anesthesia decreased the expression of PSD95 (**G**), SYN1 (**H**), and SYP (**I**). **J** Prolonged anesthesia enhanced microglial phagocytosis. The iba1(green) positive microglia were colocalized with CD68 (red) in the hippocampal CA1 region. Scale bar = 10 μm. **K** Prolonged anesthesia increased the number of microglia-colocalized CD68 (*n* = 4 per group). **L** Prolonged anesthesia enhanced microglial synaptic engulfment. The iba1(green) positive microglia were colocalized with PSD95 (red) in the hippocampal CA1 region, and microglia engulfed PSD95 puncta. Scale bar = 10 μm. **M** Prolonged anesthesia enhanced microglial chemotaxis. The iba1(green) positive microglia were colocalized with CX3CR1 (red) in the hippocampal CA1 region. Scale bar = 10 μm. **N** Prolonged anesthesia increased the number of microglia-colocalized CX3CR1 (*n* = 4 per group). **O** Microglia engulf PSD95 puncta. Scale bar = 10 μm (upper panel) or 5 μm (bottom panel). **P** Prolonged anesthesia increased the number of microglia-colocalized PSD95 (*n* = 4 per group). **Q** Prolonged anesthesia induces microglia to secrete complement. Scale bar = 10 μm. **R** Prolonged anesthesia increased the number of microglia-colocalized C1qa (*n* = 4 per group). Data was shown as Mean ± SD, with *P < 0.05 or *P < 0.001; Sevo group vs. control group. Arrows represent positive cells or colocalization

Based on the results of RNA-seq, we looked for evidence linking synaptic loss to complement-mediated microglial pruning. It has been reported that synaptic pruning by microglia continuously monitors synapses and brain development, and pathological synaptic pruning driven by complement has also been reported in many CNS diseases, including Alzheimer’s disease and frontotemporal dementia [9–11, 34]. First, we identified that synaptic markers, including PSD95 mRNA, SYN1 mRNA, and SYP mRNA in the hippocampus, were downregulated in the Sevo group compared with the control group (Fig. 5 A). Moreover, we observed that PSD95, SYN1, and SYP in the hippocampus at the protein level were also downregulated in the Sevo group compared with the control group (Fig. 5 E, G–I). Synaptic-related proteins were downregulated, which is consistent with the RNA-seq results. Then, we confirmed that complement markers, including C1qa and C3, in the hippocampus were also upregulated at both the protein and mRNA levels (Fig. 5 B, E, F).

Second, CD68, as a microglial phagocytosis marker [35], was increased in the hippocampus at both the protein and mRNA levels (Fig. 5 C–E). Moreover, the number of microglia colocalized with CD68 was increased in the hippocampal CA1 region (Fig. 5 J, K). CX3CR1 (C-X3-C motif chemokine receptor 1), expressed by both microglia and neurons, mediates microglial adhesion, chemotaxis, and migratory functions [36, 37]. Third, we found that the number of microglia colabeled with CX3CR1 was increased in the hippocampal CA1 region in the Sevo group compared with the control group (Fig. 5 M, N). Fourth, we detected that the number of microglia colabeled with PSD95 was increased in the hippocampal CA1 region after sevoflurane exposure, indicating that the microglia engulfed the PSD95 puncta (Fig. 5 L, O, P). Finally, we also observed that the number of microglia colonized with C1qa was increased, indicating that prolonged anesthesia induces microglia to produce complement C1qa (Fig. 5 Q, R). Therefore, microglia with increased phagocytotic ability, hyperactive activity, adhesion, and chemotaxis secrete complement, engulf different synapses, and cause synaptic loss and a reduction in synaptic proteins.

**Fig. 6:**
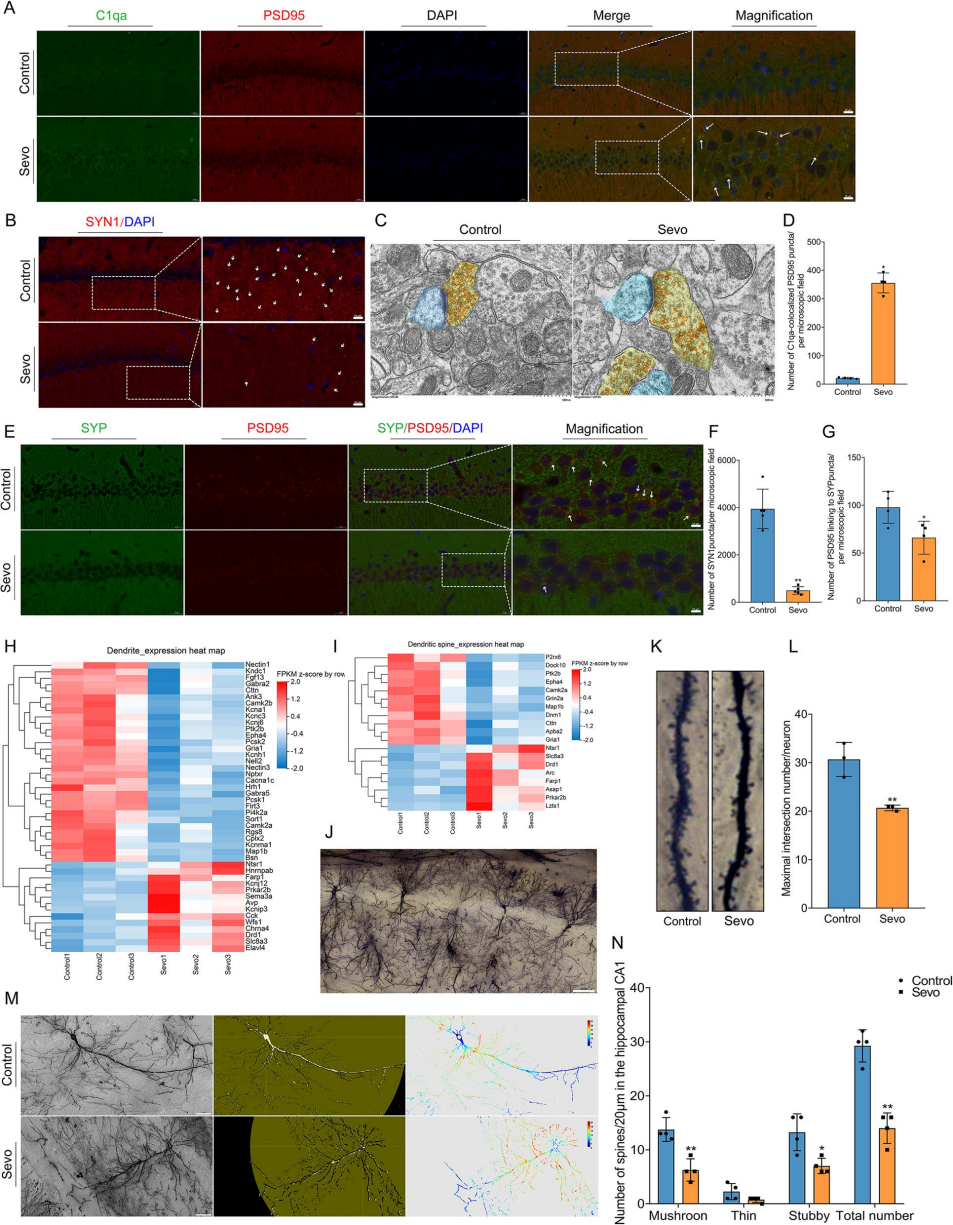
Prolonged anesthesia caused synaptic elimination in the hippocampus by complement-mediated synaptic elimination and decreased dendritic spines and their genes. **A** Prolonged anesthesia increased complement-mediated synaptic elimination in the hippocampal CA1 region. Complement C1qa (green) colocalized with PSD95 (red) puncta was elevated after prolonged anesthesia. Scale bar = 10 μm. **B** Prolonged anesthesia reduced synaptic density in the hippocampal CA1 region. SYN1 (red), as a marker of synaptic density, was sharply decreased after prolonged anesthesia. Scale bar = 10 μm. **C** SEM (scanning electron microscope) identifies ultrastructural alteration of synapses in the hippocampal. Synaptic vesicles were reduced, and postsynaptic membrane continuity was interrupted after prolonged anesthesia. Scale bar = 500 nm. **D** The number of C1qa-colocalized PSD95 between both groups (*n* = 4 per group). **E** Prolonged anesthesia reduced the number of synapses. The synapse was marked by presynaptic (SYP) and postsynaptic protein (PSD95). Synapse partially enlarged as shown in panel **E**. Scale bar = 10 μm. **F** Synaptic density in the hippocampal CA1 region between both groups (*n* = 5 per group). **G** The number of synapses in the hippocampal CA1 region between both groups (*n* = 4 per group). **H, I** Prolonged anesthesia downregulated genes of the dendrite (**H**) and dendritic spines (**I**). In panels **H** to **I**, the horizontal axis is the *z*-score of the sample, and the vertical axis is the gene. The warmer the color block is, the higher the expression level is, and the colder the color block is, the lower the expression level is (*n* = 3 per group). **J** Golgi staining in the hippocampal CA1 region. Scale bar = 100 μm. **K** Prolonged anesthesia triggered the loss of dendritic spines. **L** Maximal intersection number per neuron between groups (*n* = 3 per group). **M** Sholl analysis discovered that prolonged anesthesia decreased maximal intersection number per neuron between groups. Scale bar = 50 μm. **N** The number of dendritic spines in the hippocampus between groups (*n* = 4 per group). Data was shown as Mean ± SD, with *P < 0.05 or *P < 0.001; Sevo group vs. control group. Arrows represent synaptic puncta or colocalization

It has been widely reported that C1qa and C3 are involved in synapse loss, tagging inappropriate synaptic connections between neurons for removal by phagocytic microglia during the process of synaptic pruning [38, 39]. In the hippocampal CA1 region, the number of C1qa colabeled with PSD95 puncta increased in the Sevo group compared with the control group (Fig. 6 A, D), which suggests sevoflurane exposure induces complement-mediated synaptic pruning. We employed IF staining to examine the number of synapses and synaptic density. PSD95 (a marker for postsynaptic protein), SYN1 (a marker for presynaptic protein), and SYP (a marker for presynaptic protein) were used to detect the number of synapses and the synaptic density [40]. Synaptic density (SYN1 puncta) was reduced in the hippocampal CA1 region in the Sevo group compared with the control group (Fig. 6 B, F).

Furthermore, the number of synapses (PSD95 linked to SYP puncta) was also reduced in the hippocampal CA1 region after sevoflurane exposure (Fig. 6 E, G). SEM was used to observe the ultrastructural alterations of the synapse to detect the synaptic vesicles, presynaptic and postsynaptic membrane, active zone, and synaptic cleft. In the Sevo group, synaptic vesicles were reduced, and postsynaptic membrane continuity was interrupted (as shown in Fig. 6 C). Thus, the decrease in synaptic number and density and synaptic ultrastructural alteration responded to complement-mediated microglial pruning.

### Prolonged anesthesia reduces the number of dendritic spines and dendrites and downregulates their related genes

According to the RNA-seq results, we found that DEGs enriched in dendritic spines and dendrites were mostly downregulated (Fig. 6 H, I, and Fig. 4 B). This apparently indicated that dendritic spines and dendrites suffer from disruption after sevoflurane exposure. We used Golgi staining and Sholl analysis to detect structural alterations in the dendritic spines and dendrites in the hippocampal CA1 region (Fig. 6 J). The number of dendritic spines (mushroom, stubby, thin, and total) was reduced in the Sevo group compared with the control group (Fig. 6 K, N). In the Sholl analysis, the maximal intersection number of a neuron in the Sevo group was lower than that in the control group (Fig. 6 L, M). An intersection mask chart is shown to describe the spatial distribution and number of dendrites and dendritic spines (Fig. 6 M).

### Microglial depletion rescues cognitive dysfunction and ameliorates anxiety-like behaviors in SIN rats

**Fig. 7:**
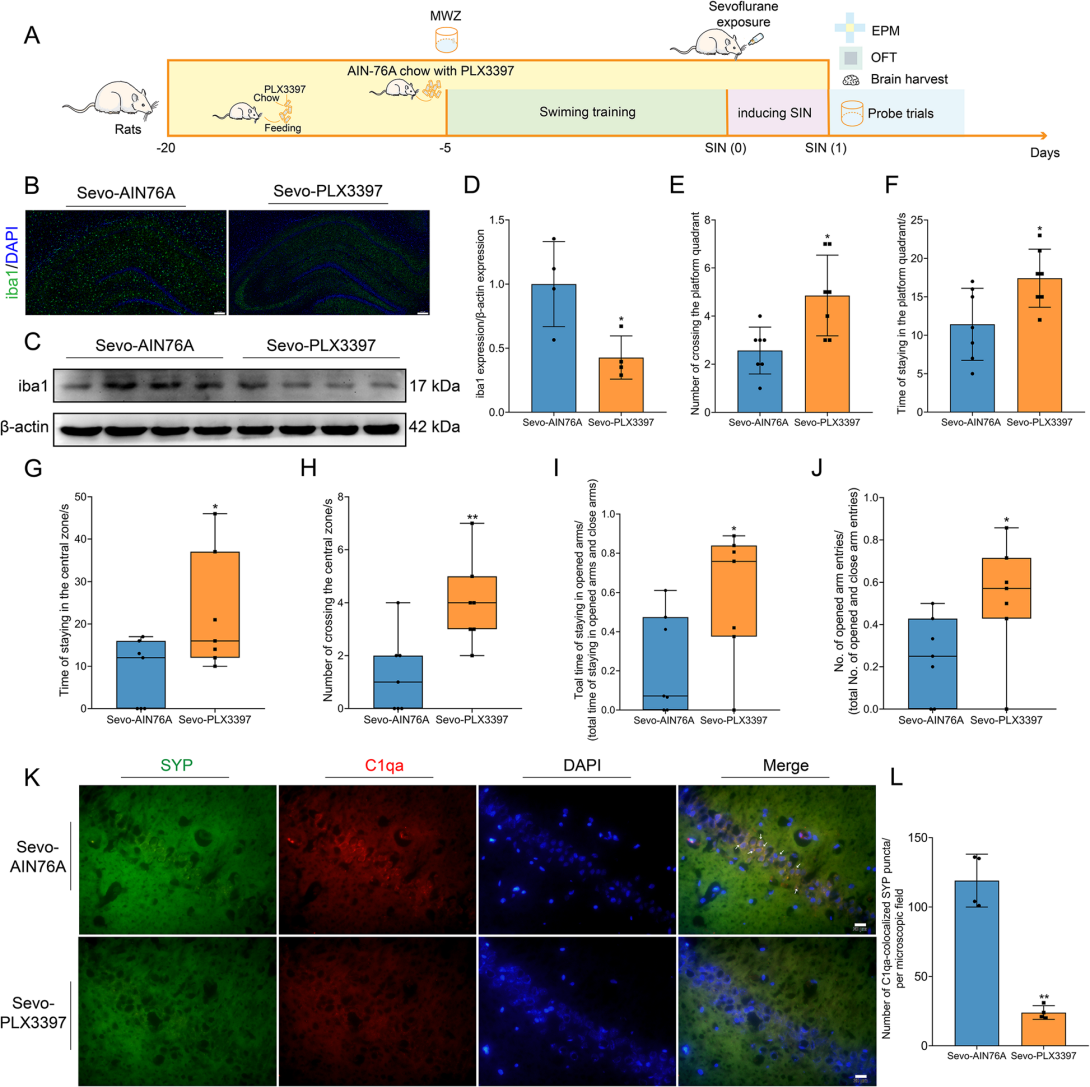
Microglial depletion rescued cognitive dysfunction and ameliorated anxiety-like behaviors in SIN rats. **A** The schedule of the second experiment. We used chow with PLX3397 to feed rats for 21 days, depleting the microglial population. **B** iba1 (green) positive microglia were sharply depleted in the hippocampus after PLX3397 treatment. Scale bar = 200 μm. **C** PLX3397 treatment effectively eliminated microglial marker-iba1 expression in the hippocampus. **D** Statistical chart of iba1 expression between both groups (*n* = 4 per group). **E, F** Microglial depletion rescued cognitive dysfunction of SIN rats (*n* = 7 per group). In MWM, microglial depletion increased the number and time of entering the platform quadrant of SIN rats. **G, H** Microglial depletion ameliorated anxiety-like behaviors of SIN rats (*n* = 7 per group). In the OPF test, microglial depletion increased the time and number of entering the central zone of SIN rats. **I, J** Microglial depletion ameliorated anxiety-like behaviors of SIN rats (*n* = 7 per group). In the EPM test, microglial depletion increased the time and number of entering the opened arms of SIN rats. **K, L** Microglial depletion decreased the number of C1qa-colocalized SYP (*n* = 4 per group). Scale bar = 20 μm. Data was shown as Mean ± SD or median ± IQR, with *P < 0.05 or *P < 0.001; Sevo-AIN76A group vs. Sevo-PLX3397. Arrows represent colocalization

Next, we examined whether hyperactive microglia could play a key role in cognitive dysfunction and anxiety-like behaviors. The schedule of the second experiment is displayed in Fig. 7 A. We took advantage of a diet supplemented with PLX3397 to feed rats to eliminate the microglial population without inducing significant cognitive damage to normal animals [12]. Thus, we fed the rats with AIN-76A chow that contains PLX3397. PLX3397 treatment effectively reduced the microglial count and iba1 protein levels in the hippocampus (Fig. 7 B–D). In the MWM test, PLX3397 treatment increased the number of crossings and time spent swimming in the platform quadrant, rescuing the cognitive impairment caused by sevoflurane exposure (Fig. 7 E, F). In the OFT, PLX3397 treatment increased the number of entries and the time spent in the central zone, ameliorating anxiety and stress (Fig. 7 G, H). In the EPM test, we also found increased open arms stay times and number of entries into the opened arms, again ameliorating anxiety-like behaviors (Fig. 7 I, J). Surprisingly, after microglial depletion, the complement colocalized with SYP puncta was sharply reduced, meaning microglial depletion ameliorated the pathological synaptic pruning driven by complement (Fig. 7 K, L).

### RNA-seq analysis shows that microglial depletion rescues the downregulation of synapse-related genes in the hippocampus

**Fig. 8:**
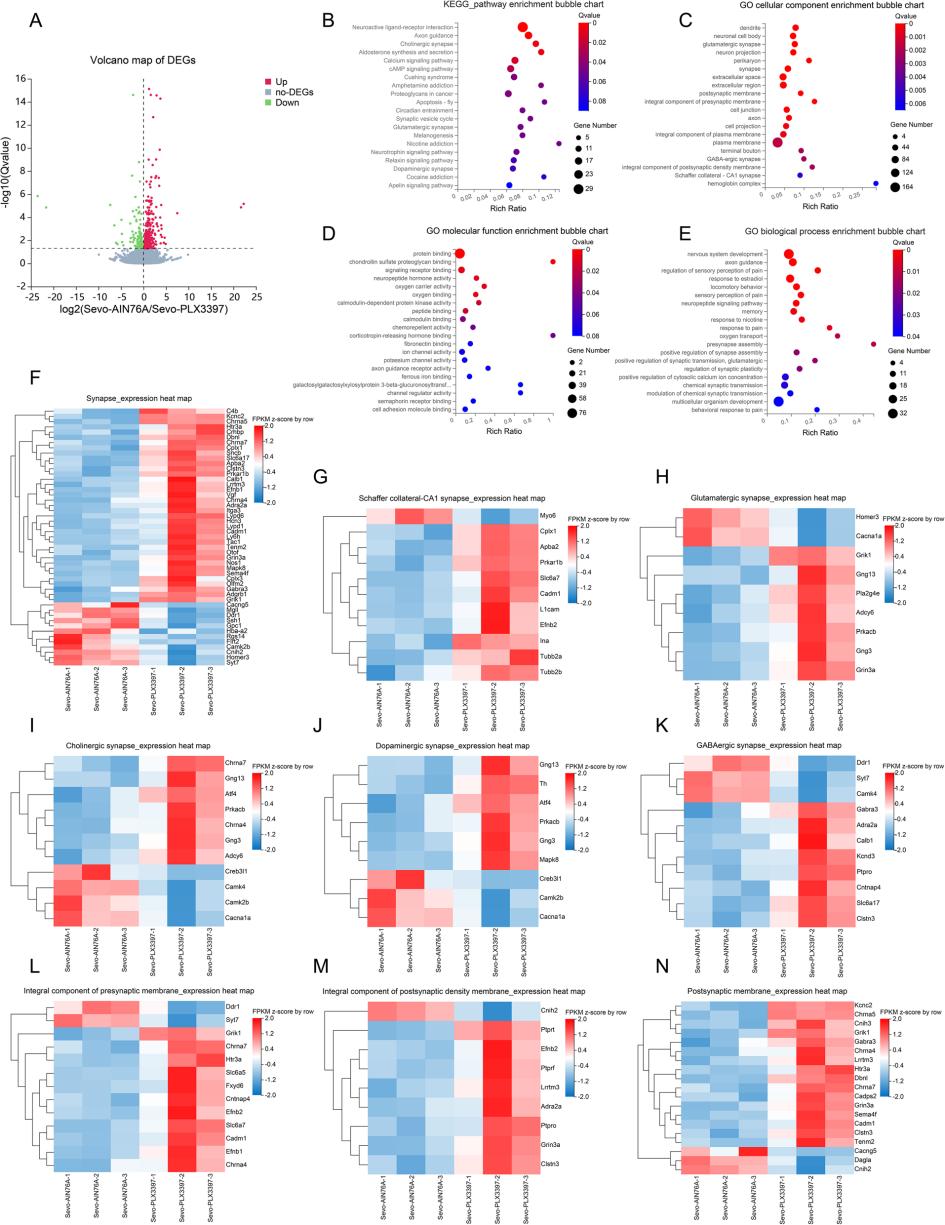
RNA-seq analysis indicated DEGs in the hippocampus enriched in different synaptic pathways, and synapse-related genes were upregulated after microglial depletion. **A** Volcano map of DEGs. The *X*-axis represents the fold change of the difference after conversion to log_2_, and the *Y*-axis represents the significance value after conversion to −log_10_. The red represents 347 DEGs upregulated, the green represents 160 DEGs downregulated, and the gray represents non-DEGs. **B** KEGG pathway enrichment bubble chart. **C** GO cellular component enrichment bubble chart. **D** GO molecular function enrichment bubble chart. **E** GO biological process enrichment bubble chart. From panels **B** to **E**, *X*-axis is the enrichment ratio (the ratio of the number of genes annotated to an entry in the selected gene set to the total number of genes annotated to the entry in the species), and *Y*-axis is KEGG pathway or GO term. The size of the bubble represents the number of genes annotated to the KEGG pathway or GO term. The color represents the enriched significance (*n* = 3 in each group). **F–K** Expression heat map of DEGs enriched in the synapse (**F**), Schaffer collateral-CA1 synapse (**G**), glutamatergic synapse (**H**), cholinergic synapse (**I**), dopaminergic synapse (**J**), and GABAergic synapse (**K**). **L–N** Expression heat map of DEGs enriched in integral component of presynaptic membrane (**L**), integral component of postsynaptic density membrane (**M**), postsynaptic membrane (**N**). From panels **F** to **N**, the horizontal axis is the *z*-score of the sample, and the vertical axis is the gene. The warmer the color block is, the higher the expression level is, and the colder the color block is, the lower the expression level is (*n* = 3 per group)

To examine whether defective microglia rescue synaptic elimination, we used RNA-seq again to identify DEGs and their enrichment (Fig. 8 A). As expected, these DEGs and enrichment pathways were also associated with synapses. In detail, the KEGG pathway enrichment chart showed that many DEGs were enriched in cholinergic synapses, synaptic vesicle cycles, glutamatergic synapses, and dopaminergic synapses (Fig. 8 B). The GO cellular component enrichment chart also showed that many DEGs were enriched in glutamatergic synapses, synapses, postsynaptic membranes, integral components of presynaptic membranes, GABAergic synapses, integral components of postsynaptic density membranes, and Schaffer collateral-CA1 synapses (Fig. 8 C). The GO molecular function enrichment chart showed that many DEGs were enriched in protein binding, signaling receptor binding, and neuropeptide hormone activity (Fig. 8 D). Then, the GO biological process enrichment chart showed that numerous DEGs were enriched in nervous system development, neuropeptide signaling pathways, memory, presynapse assembly, positive regulation of presynapse assembly, positive regulation of synaptic transmission, regulation of synaptic plasticity, and modulation of chemical synaptic transmission (Fig. 8 E).

To identify the expression level of synapse-related genes, we employed a DEG expression heatmap to observe the different types of synaptic genes. DEGs enriched in the synapse, Schaffer collateral-CA1 synapse, glutamatergic synapse, cholinergic synapse, dopaminergic synapse, and GABAergic synapse were predominantly upregulated (Fig. 8 F–K). DEGs of synaptic components, including integral components of the presynaptic membrane, integral components of the postsynaptic density membrane, and postsynaptic membrane, were also upregulated (Fig. 8 L–N). Therefore, PLX3397 treatment rescues the loss of synapse-related genes and exerts positive regulation of presynapse assembly, synaptic transmission, synaptic plasticity, and chemical synaptic transmission.

### Microglial depletion ameliorates activation of the NF-κB inflammatory pathway, hippocampal inflammation, complement activation, and synaptic elimination

**Fig. 9:**
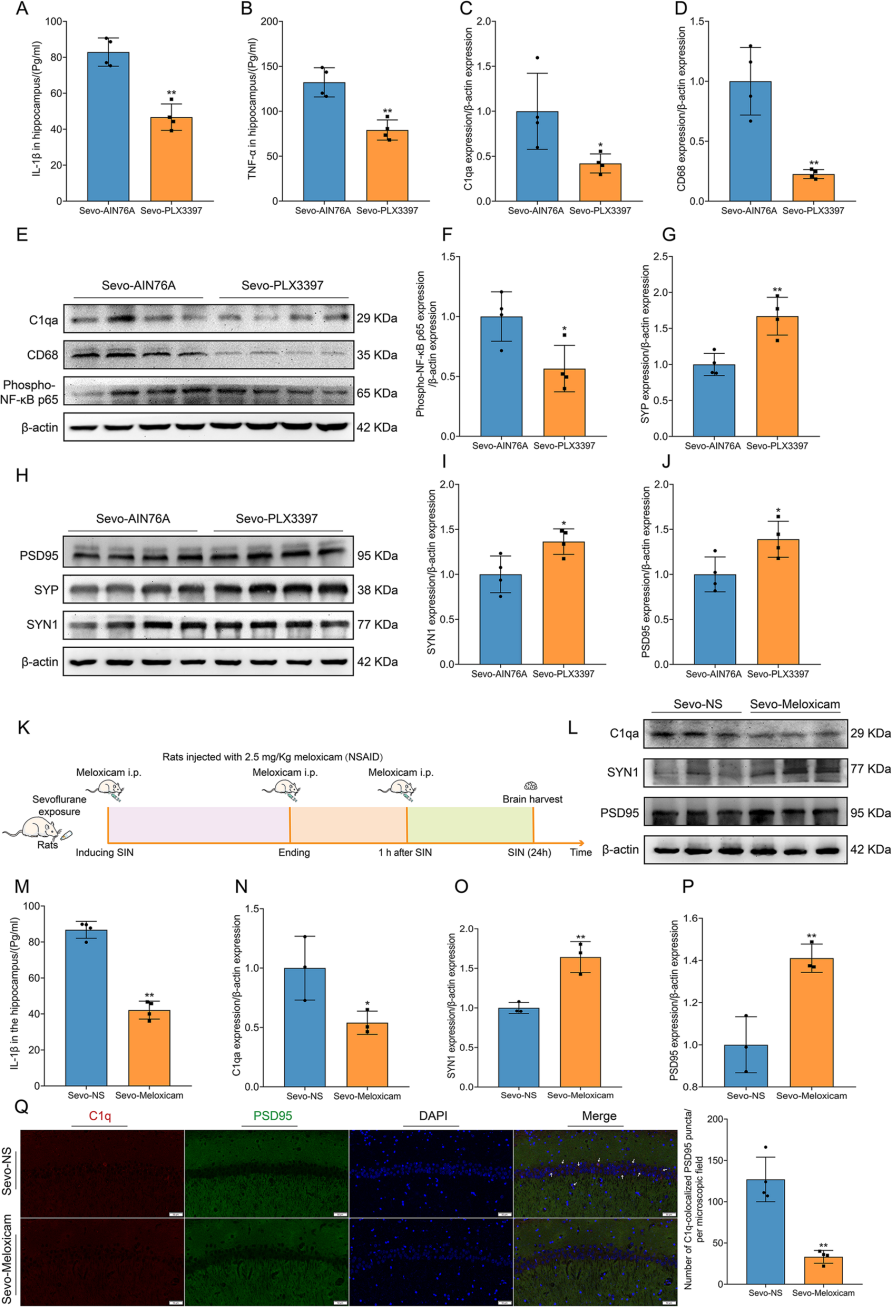
Microglial depletion ameliorated the activation of the NF-κB inflammatory pathway, hippocampal inflammation, complement activation, and synaptic elimination, and neuroinflammatory inhibition also rescued synaptic elimination. **A, B** Microglial depletion reduced the level of IL-1β (**A**) and TNF-α (**B**) in the hippocampus (*n* = 4 per group). **C** Microglial depletion decreased the expression of C1qa in the hippocampus (*n* = 4 per group). **D** Microglial depletion decreased the expression of CD68 in the hippocampus (*n* = 4 per group). **E** Immunoblotting band of C1qa, CD68, Phospho-NF-κB p65, and β-actin. **F** Microglial depletion reduced the expression of Phospho-NF-κB p65 in the hippocampus (*n* = 4 per group). **G, H** Microglial depletion increased the expression of SYP in the hippocampus (*n* = 4 per group). Microglial depletion rescued synaptic loss in the hippocampus. **I, J** Microglial depletion increased the expression of SYN1 (**I**) and PSD95 (**J**) in the hippocampus (*n* = 4 per group). **K** The schedule of the third experiment. Intraperitoneal injection of meloxicam was administered three times: at the time of sevoflurane exposure, at the time of anesthesia end, and 1 h after anesthesia end. **L** Neuroinflammatory inhibition also reduced complement and rescued synaptic elimination. **M** Neuroinflammatory inhibition decreased the level of IL-1β in the hippocampus (*n* = 4 per group). **N** Neuroinflammatory inhibition decreased C1qa expression in the hippocampus (*n* = 3 per group). **O, P** Neuroinflammatory inhibition increased SYN1 (**O**) and PSD95 (**P**) expression in the hippocampus (*n* = 3 per group). **Q** Suppression of neuroinflammation inhibiting complement-mediated synaptic loss (*n* = 4 per group). Scale bar = 50 μm. Data was shown as Mean ± SD, with *P < 0.05 or *P < 0.001; Sevo-NS group vs. Sevo-Meloxicam group. Arrows represent colocalization

We then investigated whether PLX3397 treatment could disrupt microglial phagocytosis, the inflammatory response, complement production, and synaptic loss. ELISA showed that the proinflammatory cytokines IL-1β and TNF-α were reduced in the hippocampus (Fig. 9 A, B). Furthermore, PLX3397 treatment significantly inhibited the NF-κB inflammatory pathway by downregulating phospho-NF-kB p65 expression (Fig. 9 E, F). In addition, PLX3397 treatment suppressed the phagocytosis of microglia (downregulation of CD68 expression) and complement production (Fig. 9 C, D). Then, microglial depletion by PLX3397 treatment rescued synaptic elimination and restored the expression of synaptic proteins (Fig. 9 G–J). Accordingly, defective microglia induced by PLX3397 prevented microglia-mediated pathophysiological effects, including neuroinflammation, complement production, synaptic pruning, and synaptic elimination.

### Neuroinflammatory inhibition rescues synaptic elimination and suppresses complement activation

The therapeutic effect of microglial elimination and neuroinflammatory inhibition rescues synaptic loss. However, it was unclear whether only inhibiting neuroinflammation could reverse the synaptic loss. We applied the NSAID-meloxicam to diminish neuroinflammation and detected the effect of anti-neuroinflammation on synaptic loss. The schedule of the third experiment is displayed in Fig. 9 K. At first, meloxicam can effectively inhibit neuroinflammation in the hippocampus (Fig. 9 M). Surprisingly, neuroinflammatory inhibition also reduces synaptic elimination, decreases complement activation, and rescues the loss of synaptic proteins (Fig. 9 L–P). Meanwhile, the number of C1q colabeled with PSD95 sharply decreased after neuroinflammatory suppression (Fig. 9 Q). Hence, neuroinflammatory suppression through inhibiting complement-mediated synaptic loss reversed prolonged anesthesia-induced pathologically synaptic impairment.

### C1q neutralization inhibits complement-mediated synaptic elimination and suppresses microglial activation, which ameliorates cognitive dysfunction and anxiety-like behaviors in SIN rats

**Fig. 10:**
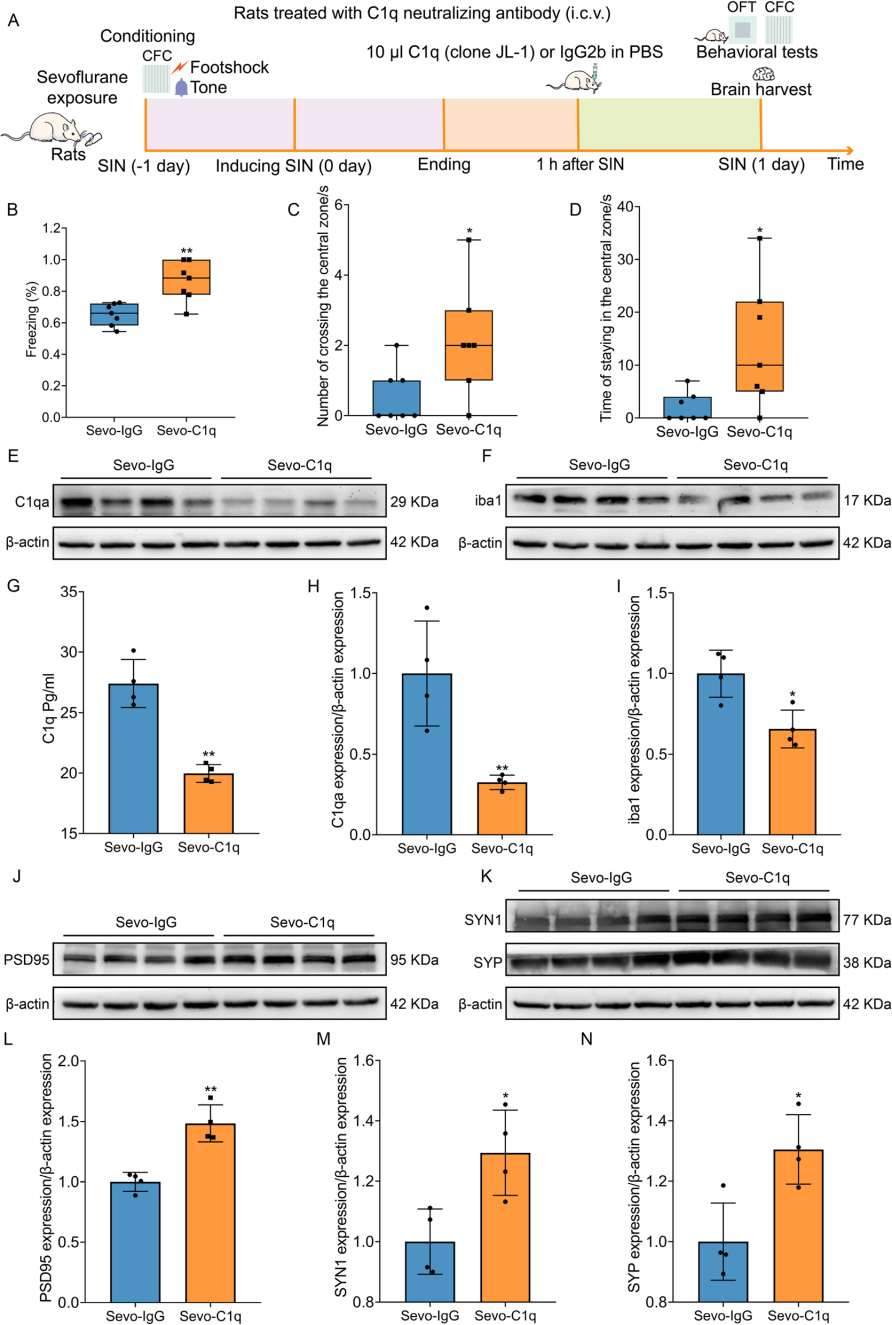
C1q neutralization also rescued synaptic elimination, inhibited microglial activation, and ameliorated cognitive dysfunction and anxiety-like behaviors in SIN rats. **A** The schedule of the fourth experiment. Intracerebroventricular (i.c.v) injection of 10 μl C1q neutralizing antibody or IgG2b in PBS was administered at 1 h after anesthesia end. CFC and OFT were used to estimate the behaviors of rats. **B** The percentage of freezing time of rats during the CFC test (*n* = 7 per group). **C, D** C1q neutralization reduced anxiety-like behaviors of rats in OFT (*n* = 7 per group). **E, F** Immunoblotting band of C1qa, iba1, and β-actin. **G** ELISA showed that C1q neutralization reduced the level of C1q in the hippocampus. **H, I** C1q neutralization downregulates the C1qa and iba1 expression in the hippocampus. **J, K** Immunoblotting band of PSD95, SYN1, SYP, and β-actin. **L–N** C1q neutralization increased the level of PSD95 (**L**), SYN1 (**M**), and SYP (**N**) in the hippocampus. Data was shown as Mean ± SD or median ± IQR, with *P < 0.05 or *P < 0.001; *n* = 4 per group, Sevo-IgG group vs. Sevo-C1q group

The schedule of the fourth experiment is displayed in Fig. 10 A. We applied the C1q neutralizing antibody to diminish C1q and detected the effect of complement on synaptic loss. ELISA showed that the C1q neutralizing antibody can effectively reduce the level of C1q in the hippocampus (Fig. 10 G). WB analysis also confirmed that C1q neutralizing antibody sharply downregulated C1qa expression in the hippocampus (Fig. 10 E, H). In the CFC, C1q neutralization increased the percentage of freezing time, rescuing the cognitive impairment caused by sevoflurane exposure (Fig. 10 B). In the OFT, C1q neutralization increased the number of entries and the time spent in the central zone, ameliorating anxiety and stress (Fig. 10 C, D). Along with C1q neutralization, iba1 expression was downregulated (Fig. 10 F, I). Meanwhile, the expression of synaptic proteins (PSD95, SYN1, and SYP) was also increased in the hippocampus (Fig. 10 J–N). Therefore, C1q neutralization can inhibit complement-mediated synaptic elimination and suppress microglial activation, rescuing synaptic loss, which ameliorates cognitive dysfunction and anxiety-like behaviors in SIN rats.

## Discussion

Our results illustrated that prolonged anesthesia in rats causes cognitive dysfunction, anxiety-like behaviors, neuroinflammation, and complement activation. We identified critical microglial synaptic elimination driven by complement and neuroinflammation in the hippocampus after prolonged anesthesia. Using multiple experimental methods, we found that there was a remarkable increase in phagocytic and chemotactic microglia, accumulation of C1qa on synapses, and downregulation of synaptic proteins and genes in the hippocampus. Activated microglia with a specific morphological structure and phenotype induce neuroinflammation and complement activation, engulf synapses, and cause synaptic loss. Depletion of microglia by PLX3397 treatment blocks this process. Furthermore, inhibiting neuroinflammation with meloxicam and C1q neutralization also rescued synaptic loss.

Our findings suggest that hippocampal neuroinflammation and complement activation inducing pathological synaptic pruning mediated by microglia is a key mechanism underlying prolonged anesthesia-induced synaptic elimination. C1qa, as a main component of C1q, was aberrantly increased in the hippocampus and deposited onto synapses, activating the downstream classical complement pathway (C3) and triggering an increase in phagocytic microglia. Through the C1qa and C3 “Eat Me” complement pathways, microglia modulate synaptic pruning [41]. Complement factors C1q and C3 label subsets of localized synapses for removal by phagocytic microglia [9]. Our data also found that C1qa binds synapses mediating the “Eat Me” pathway and driving microglia to engulf different types of synapses, leading to a loss of synapses and dendritic spines. RNA-seq confirmed that the DEGs were primarily enriched in different types of synapses (glutamatergic, cholinergic, dopaminergic, and GABAergic synapses). Gene expression in these synapses was also downregulated in response to prolonged anesthesia triggering synaptic pruning. Complement-mediated microglial pruning has been reported to be involved in different CNS diseases, including Alzheimer’s disease, autism, frontotemporal dementias, schizophrenia, and epilepsy, and it occurs in different regions, including the hippocampus, cerebellum, barrel cortex, and visual system [9, 10, 42–46]. Similarly, our study revealed that prolonged anesthesia caused complement-mediated microglial pruning in the hippocampus, participating in synaptic loss-induced cognitive dysfunction and anxiety-like behaviors, a newly described mechanism underlying neurocognitive impairment in SIN models.

The role of neuroinflammation and the microglia-mediated inflammatory response in inducing SIN has been demonstrated by numerous studies [47–49]. Pathological synaptic pruning mediated by microglia can also be activated in response to inflammation, as reported in West Nile virus infection, sepsis-associated encephalopathy, and lupus [38, 50, 51]. Complement expression is altered according to neuroinflammation. In response to inflammation, increased levels of complement proteins are observed in the cerebrospinal fluid [50]. In systemic inflammation induced by lipopolysaccharide (LPS), it has been reported that a phagosomal inflammatory response of microglia leads to complement-mediated synaptic pruning, resulting in the loss of dopaminergic neurons [52]. In addition, in traumatic brain injury, the chronic neuroinflammatory response is associated with continued complement activation and it promotes microglial phagocytosis of complement-opsonized synapses [53]. Complement inhibition interrupted the degenerative neuroinflammatory response and reversed synaptic loss [53]. Thus, there is a close link between complement and neuroinflammation in mediating synaptic pruning. In our study, prolonged anesthesia triggered sharply increased neuroinflammation and complement activation in the hippocampus. Next, complement C1qa and C3 continued to increase in response to the neuroinflammatory response, ultimately triggering microglia-mediated synaptic pruning and synaptic elimination. When we used meloxicam to inhibit hippocampal neuroinflammation or C1q neutralization, complement cascades, microglial activation, and synaptic loss were interrupted. Hence, complement interacts with the neuroinflammatory response, participating in synaptic loss, and neuroinflammatory inhibition or C1q neutralization rescues synaptic loss. These findings have direct therapeutic relevance for PND patients.

Our experiments discovered that resident microglia in the hippocampus phagocytose different synapses when challenged by prolonged anesthesia, implicating that microglia is an essential cellular mediator of synaptic elimination. Other studies also confirmed the role of microglia in engulfing synapses in some CNS diseases [9, 10]. Depletion of microglia has been previously demonstrated to effectively block complement-mediated synaptic pruning and synaptic loss [11, 54, 55]. Similarly, our study also found that the depletion of microglia induced by PLX3397 treatment was sufficient to reverse synaptic elimination, cognitive impairment, and anxiety-like behaviors caused by prolonged anesthesia. RNA-seq also confirmed that the elimination of microglia upregulated different types of synaptic genes and synaptic component genes, reflecting the therapeutic effect of microglial depletion. In addition, the depletion of microglia highly ameliorated the neuroinflammatory response and complement activation in the hippocampus. By decreasing the production of complement and neuroinflammation, “Eat Me” complement pathway-mediated synaptic loss was also interrupted, which is another therapeutic effect of microglial depletion. Together, microglia and immune-related pathways act as critical mediators of synaptic elimination, cognitive dysfunction, and anxiety-like behaviors.

Sevoflurane exposure can promote microglial migration, activation, proliferation, and phagocytic efficiency [56]. Furthermore, in isolated microglia, sevoflurane exposure resulted in increased levels of TNF-α, IL-6, and IL-1β via the NF-κB pathway [57, 58]. In addition, sevoflurane can enhance the microglial proinflammatory phenotype (M1) and simultaneously suppress the anti-inflammatory phenotype (M2 )[59]. Consistent with previous studies, our study also found that prolonged anesthesia by sevoflurane exposure activates the NF-κB pathway, increasing proinflammatory microglia, and neuroinflammatory responses and altering the microglial morphology and function in the hippocampus. When challenged by sevoflurane-induced pathological changes, microglia and complement are activated and involved in synaptic engulfment and neurobehavioral alteration. Meanwhile, microglial synaptic engulfment may be enhanced in response to the neuroinflammatory response. The reason for this is that inflammatory cytokines such as TNF-α can reactivate microglia by a positive feedback mechanism [60]. After inhibition of the neuroinflammation, the microglial synaptic loss was disrupted. Thus, sevoflurane exposure directly triggered microglial functional changes and neuroinflammation, and the neuroinflammatory response reactivated microglia and complement, which mediated synaptic loss.

The neurotoxicity of sevoflurane in aged rats was observed in our study. Sevoflurane can be protective or toxic depending on the age and pathological condition of the brain. Specifically, for the adult brain, sevoflurane presents protective effects in various neurological disorders, such as traumatic, hypoxic, or ischemia-reperfusion injuries. Conversely, sevoflurane produces neurotoxicity for neonatal and elderly patients even if the brain is under normal conditions. The reasons for this discrepancy are not fully understood. Some studies attributed this disparity to different BBB permeability in response to sevoflurane exposure between the two age groups [61, 62]. In addition, different neuronal cell apoptosis, TGF-β/Smad-related pathways, and inflammatory responses after sevoflurane exposure have also been reported to be associated with the observed differences [63].

RNA-seq identified genes of different types of synapses, including glutamatergic, cholinergic, dopaminergic, and GABAergic synapses that were downregulated by sevoflurane exposure. Eliminating microglia rescued the loss of synaptic-related genes in these synapses. It seems that microglia-mediated synaptic elimination has no selection specificity. A limitation of this study was not examining the influence of microglia-mediated synaptic pruning on different types of synapses. Although we showed that glutamatergic, cholinergic, dopaminergic, and GABAergic synapses were all influenced by prolonged anesthesia, each type of synaptic-specific change in response to prolonged anesthesia is still unknown. It will be important in future work to answer this question. It has been indicated that iba1 can be a marker for microglia or infiltrated macrophages. The distinction between microglia and infiltrated macrophages (rarely in CNS) was not performed in this study.

## Conclusions

**Fig. 11:**
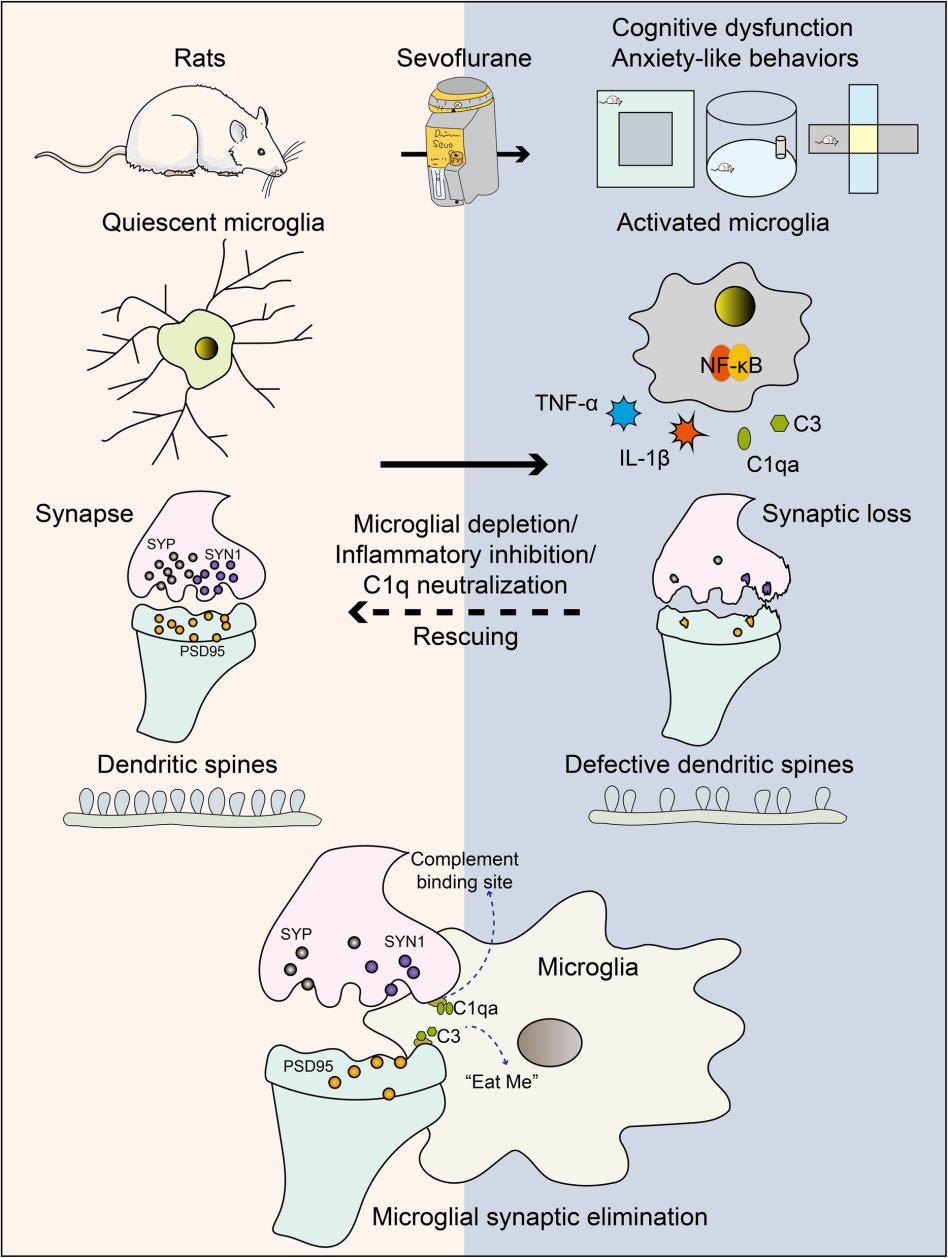
The pathogenesis diagram of the SIN. Prolonged anesthesia by sevoflurane in rats triggers cognitive impairment, anxiety-like behaviors, neuroinflammation, and complement activation. We observed critical microglial synaptic elimination and dendritic spines loss driven by complement and neuroinflammation in the hippocampus after prolonged anesthesia. There was a remarkable increase in phagocytic and chemotactic microglia, accumulation of C1qa on synapses, and downregulation of synaptic proteins and genes in the hippocampus. Activated microglia with a specific morphological structure and phenotype induce neuroinflammation and complement activation, engulf synapses, and cause synaptic loss. Depletion of microglia by PLX3397 treatment blocks this process. Furthermore, inhibiting neuroinflammation with meloxicam and C1q neutralization also rescued synaptic loss

Finally, this study demonstrated that prolonged anesthesia triggers neuroinflammation-mediated and complement-mediated microglial synaptic elimination, causing neurocognitive dysfunction and anxiety-like behaviors. We showed that the depletion of microglia, inhibition of neuroinflammation, or C1q neutralization prevented pathological synaptic loss. Our work provides new insights into how cognitive functions are impaired in SIN and what the neuromorphopathological underpinnings of SIN are, which is valuable for preventing PND. The pathogenesis diagram of the SIN is shown in Fig. 11.

## Supplementary information

**Additional file 1:.** Table. S1: Primers of RT-PCR.

**Additional file 2: **Fig. S1: ELISA used to detect peripheral inflammation in serum. Data was shown as Mean ± SD, with *P < 0.05 or *P < 0.001; n = 6 per group, Sevo group vs. control group.

## Data Availability

All data generated or analyzed during this study are included in this published article.

## Abbreviations

Arg1: Arginase-1
BBB: Blood-brain barrier
BSA: Bovine serum albumin
BSR: Burst suppression ratio
CFC: Contextual fear conditioning
CNS: Central nervous system
CX3CR1: C-X3-C motif chemokine receptor 1
DEGs: Differently expressed genes
ELISA: Enzyme-linked immunosorbent assay
EPM: Elevated plus maze
FPKM: Fragments per kilobase per million reads
GO: Gene Ontology
HE: Hematoxylin and eosin
i.c.v.: Intracerebroventricular
iNOS: Inducible nitric oxide synthase
IQR: Interquartile range
KEGG: Kyoto Encyclopedia of Genes and Genomes
LFP: Local field potential
MWM: Morris water maze
NS: Normal saline
NSAID: Nonsteroidal anti-inflammatory drug
OFT: Open field test
PBS: Phosphate-buffered saline
PND: Perioperative neurocognitive disorders
RNA-seq: RNA sequencing
RT-PCR: Real-time polymerase chain reaction
SD: Sprague Dawley
SD: Standard deviation
SDS-PAGE: Sodium dodecyl sulfate-polyacrylamide gel electrophoresis
SEM: Scanning electron microscope
Sevo: Sevoflurane
SIN: Sevoflurane-induced neurotoxicity
SYN1: Synapsin-1
SYP: Synaptophysin
