# Supplementary material for: Prolonged anesthesia induces neuroinflammation and complement-mediated microglial synaptic elimination involved in neurocognitive dysfunction and anxiety-like behaviors
Source: BMC Med. 2023 Jan 5;21:7. doi: 10.1186/s12916-022-02705-6 (PMC9814183; doi:10.1186/s12916-022-02705-6)
Supplement: Supplementary file 1 — Additional file 1:. Table. S1: Primers of RT-PCR. [file 12916_2022_2705_MOESM1_ESM.docx]

| Additional file 1:Table. S1: Primers of RT-PCR | | |
| --- | --- | --- |
| RNA | Sequences forward/reversed 5`-3` | |
| β-actin | Forward | CGTTGACATCCGTAAAGACCTC |
|  | Reversed | TAGGAGCCAGGGCAGTAATCT |
| SYP | Forward | TGGGTTGGCAACTTATGGTTC |
|  | Reversed | TAATCGGGTTGATAACCACCC |
| SYN1 | Forward | CTTCTCCTCGCTGTCTAACGC |
|  | Reversed | GCATGAGCCACAAGATTGAGAT |
| PSD95 | Forward | CAAGAAATACCGCTACCAAGATG |
|  | Reversed | ATCTCCCCCTCTGTTCCATTC |
| IL-4 | Forward | CTCCGTGCTTGAAGAACAAGTC |
|  | Reversed | CAGTGTTGTGAGCGTGGACTC |
| IL-6 | Forward | TTCTCTCCGCAAGAGACTTCC |
|  | Reversed | GTGGGTGGTATCCTCTGTGAAG |
| IL-10 | Forward | GCAGGACTTTAAGGGTTACTTGG |
|  | Reversed | ATCATTCTTCACCTGCTCCACT |
| IL-1β | Forward | GTGGCAGCTACCTATGTCTTGC |
|  | Reversed | CCACTTGTTGGCTTATGTTCTGT |
| TNF-α | Forward | TGATCGGTCCCAACAAGGAG |
|  | Reversed | GGTTGTCTTTGAGATCCATGCC |
| iNOS | Forward | AAACAACAGGAACCTACCAGCTC |
|  | Reversed | CACTGTTAGTGGCGTAAAGTATGTG |
| Arg1 | Forward | ATTGGCAAAGTGATGGAAGAGAC |
|  | Reversed | CAAGACAAGGTCAACGCCAC |
| CD68 | Forward | CAGTGGACATTCTCAGCGCA |
|  | Reversed | GTAACGCAGAAGGCAATGAGC |
| CD86 | Forward | TAAGCAAGGATACCCGAAACC |
|  | Reversed | CCGGGAATGGAAGAGATAGG |
| CD206 | Forward | GCCTACTGCCTGCCCTAATC |
|  | Reversed | CCCATCGCTCCACTCAAAGT |
| C1qa | Forward | CAGGAATTCCCTTGGTTTCTG |
|  | Reversed | GATGCTGTCGGCTTCAGTACC |
| C3 | Forward | TTCTGGCAGTGAGCTTGTCC |
|  | Reversed | AGCACAGCTCTGATCTCCACC |
