# Supplementary figures and images for: Prolonged anesthesia induces neuroinflammation and complement-mediated microglial synaptic elimination involved in neurocognitive dysfunction and anxiety-like behaviors
Source: BMC Med. 2023 Jan 5;21:7. doi: 10.1186/s12916-022-02705-6 (PMC9814183; doi:10.1186/s12916-022-02705-6)

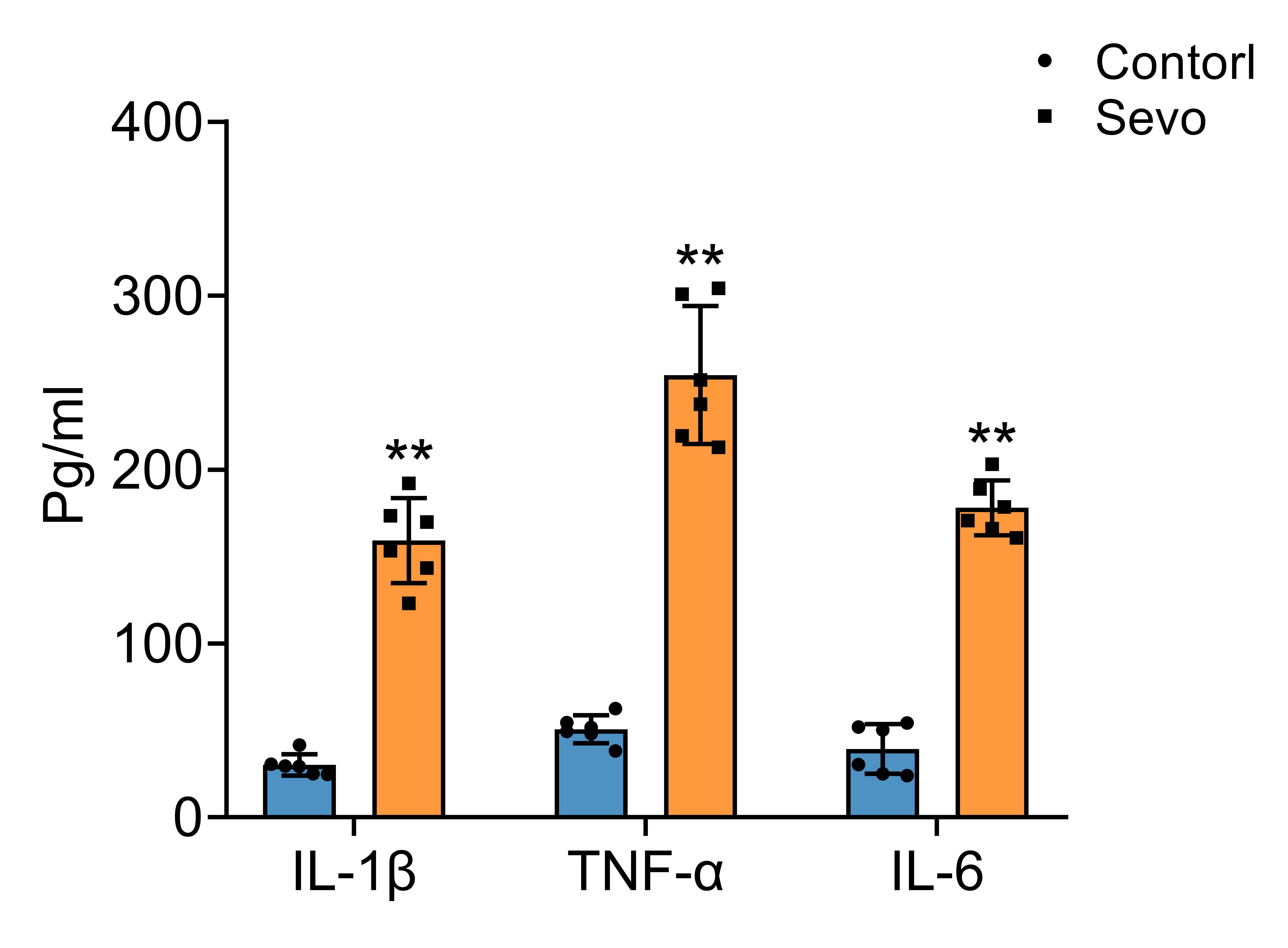

Supplement: Supplementary file 2 — Additional file 2: Fig. S1: ELISA used to detect peripheral inflammation in serum. Data was shown as Mean ± SD, with *P < 0.05 or *P < 0.001; n = 6 per group, Sevo group vs. control group. [file 12916_2022_2705_MOESM2_ESM.jpg]
